# Comprehensive Transcriptomic Analysis Reveals Prognostic Value of an EMT-Related Gene Signature in Colorectal Cancer

**DOI:** 10.3389/fcell.2021.681431

**Published:** 2021-06-15

**Authors:** Shaobo Mo, Weixing Dai, Zheng Zhou, Ruiqi Gu, Yaqi Li, Wenqiang Xiang, Lingyu Han, Long Zhang, Renjie Wang, Guoxiang Cai, Sanjun Cai, Lu Gan, Qingguo Li

**Affiliations:** ^1^Department of Colorectal Surgery, Fudan University Shanghai Cancer Center, Shanghai, China; ^2^Department of Oncology, Shanghai Medical College, Fudan University, Shanghai, China; ^3^Department of Cancer Institute, Fudan University Shanghai Cancer Center, Fudan University, Shanghai, China; ^4^Department of Medical Oncology, Zhongshan Hospital, Fudan University, Shanghai, China; ^5^Department of Cancer Center, Zhongshan Hospital, Fudan University, Shanghai, China; ^6^Center of Evidence-Based Medicine, Fudan University, Shanghai, China

**Keywords:** colorectal cancer, EMT, signature, prognosis, nomogram

## Abstract

Lymph node metastasis (LNM) is closely related to the postoperative recurrence of colorectal cancer (CRC), and greatly affects patient survival. Conducting Gene set variation analysis (GSVA) and gene set enrichment analysis (GSEA), we found that the epithelial-mesenchymal transition (EMT) signaling pathway is the signaling pathway most relevant to the process of LNM. An EMT-related gene signature was identified from a discovery dataset obtained 489 patients using LIMMA and LASSO Cox methods. Six external independent dataset analyses including a total of 1,045 CRC patients and stratification analysis showed that EMT-related gene signature could sort out those high- and low-risk CRC patients accurately. Functional analysis and loss-of-function exploration *in vitro* and *in vivo* indicated that the EMT-related-signature-associated coding genes might play functional roles in the sophisticated regulation of CRC proliferation and metastasis. Prognostic nomograms integrating the EMT-related gene signature and clinicopathological risk factors were constructed for use as numerical prediction tools to assess clinical prognosis and clinical decision-makings. The comprehensive transcriptomic analysis in this article highlights the prognostic value of an EMT-related gene signature for postoperative disease recurrence in CRC patients and reveals a potential prognostic and therapeutic biomarker for CRC.

## Introduction

The United States’ 14.8 million new colorectal cancer (CRC) cases made CRC the most common cancer of the digestive tract; with 146 deaths per day in 2019 (Siegel et al., 2020). Closely related to economic development, CRC has emerged as a critical public health problem in China as the standard of living of its people has improved; the incidence of CRC in China was approximately 37.6/100,000 in 2016, which ranks third among all types of cancer in both the United States and China (Chen et al., 2016). Colorectal resection is followed by relapse, and most such relapses occur within 2 years. The survival and quality of life of CRC patients are affected by early relapse, which includes locoregional recurrence (∼10%), distant metastasis (∼80%), and local recurrence with distant metastasis (∼10%) occurring within 2 years after primary resection (Beets-Tan and Beets, 2004). The mechanism through which early recurrence after radical resection of CRC occurs remains unclear, and the current incidence of recurrence and metastasis requires improved postoperative prediction and monitoring.

According to current National Comprehensive Cancer Network (NCCN) guidelines, lymph node metastasis (LNM) may contribute to poor survival outcomes in CRC patients, since LNM status provides valuable information about the prognosis for CRC. Epithelial-mesenchymal transformation (EMT), firstly described in the 1980s. EMT is involved in physiological embryogenesis as well as in some pathological processes, enables epithelial cells to acquire tumor characteristics such as invasive properties and LNM ability (Hanada et al., 2009). Over the course of EMT, cells lose the characteristics of epithelial and obtain certain mesenchymal properties, including motile and invasive features. EMT may enhance migration, invasion, and metastasis by tumor cells by reducing the expression of cadherin. However, these mesenchymal properties are reversible. Cells can regain epithelial characteristics through a process referred to as mesenchymal-epithelial transition (MET), enabling malignant cells to relocate to lymph metastasis (Trusolino et al., 2010). Various studies have indicated that EMT is associated with neoplastic invasion and with the progression of various cancers, including CRC (De Craene and Berx, 2013). Since EMT is a vital process in the development of CRC, it remains a subject of intense research aimed at determining whether EMT is a trigger for lymphatic invasion by CRC. In this work, we attempted to determine whether EMT is closely related to local relapse and prognosis in CRC patients.

At present, the most common risk factor for predicting the survival rates of CRC patients is based on the tumor-node-metastasis (TNM) staging system. Although the TNM staging system has been used in the evaluation of CRC patients worldwide, prognoses vary significantly due to the heterogeneity of CRC patients with similar TNM stages. The occurrence of LNM in CRC patients is associated with higher TNM stage and mortality (Kitajima et al., 2004; Suh et al., 2012), and this has inspired scientists to search for genes related to LNM, in order to get a better prediction of the survival of CRC patients. Since 2001, several genetic and mathematical models have been developed to compensate for hidden defects in the TNM staging system. Numerous studies of many malignancies have suggested that multigene expression signatures can be used to make a good prediction of cancer prognosis (Chen et al., 2011; Tan and Tan, 2011; Zhou et al., 2019). Essentially, clinical parameters from the diagnostic workup, including sex and age, can be combined with information on TNM stage and gene expression, and this information can be used to predict CRC patient survival.

In this study, using data from several cohorts in the Gene Expression Omnibus (GEO) datasets, we found that the EMT signaling pathway is the only common enriched pathway in the transition of CRC from LNM-negative to LNM-positive. Then, an EMT-related gene signature was identified and validated in CRC; the results showed that CRC patients with high EMT risk scores were very likely to have shorter disease-free survival and overall survival than CRC patients with low-risk scores. Furthermore, functional analysis demonstrated that EMT-related signature-associated coding genes are significantly enriched in cell morphogenesis, development, junctions, and critical cancer pathways that could be pivotal in CRC recurrence. In particular, a loss-of-function assay of selected genes indicated that EMT-related signature-associated coding genes might play functional roles in the sophisticated regulation of CRC proliferation and metastasis. Nomograms integrating the EMT-related gene signature and clinicopathological risk factors were constructed for use as numerical prediction tools to assess clinical prognosis and clinical decision-making.

## Materials and Methods

### Study Design and Data Collection

Epithelial-mesenchymal transition-related genes were collected from public databases [dbEMT^1^ (Zhao et al., 2015) and MSigDB^2^ ]. A detailed list of EMT-related genes is presented in Supplementary Table 1. The 50 hallmark gene sets were downloaded from the Gene Set Enrichment Analysis (GSEA) database^3^. Raw microarray CRC datasets, including GSE39582, GSE37892, GSE33113, GSE17538, and GSE14333, were downloaded from the GEO database^4^. The detailed clinical information is shown in Supplementary Table 2. Robust Multiarray Average was used to normalize the raw data (Irizarry et al., 2003). CRC cases in the TCGA dataset with clinical and gene expression information were downloaded from UCSC Xena^5^. The detailed clinical information on these cases is shown in Supplementary Table 3.

### Gene Set Variation Analysis (GSVA) and GSEA

Lymph node metastasis-positive and LNM-negative CRC groups in this study were defined from the GSE39582, GSE37892, GSE17538, and GSE14333 datasets. According to the reference gene set, hallmark gene sets, we used GSVA to calculate a signaling pathway variation score for each sample in stages I–III CRC using the “GSVA” R package (Hanzelmann et al., 2013), setting the *p*–value <0.05 as statistical significance. Using the same datasets described above, GSEA was also performed to analyze differences between CRC patients in the LNM-positive and LNM-negative subgroups via “javaGSEA” to show the common GSEA result (Subramanian et al., 2005). In GSVA and differently expressed genes (DEGs) analysis, Benjamini and Hochberg method was used to adjust *p*-value as FDR correction to ensure that FDR < 0.25.

### Construction of the Prognostic EMT-Related Gene Signature

Patients who experienced disease recurrence within 2 years after primary resection were classified into the early relapse group. Long-term survival refers to no relapse after a minimum of 5 years of follow-up. We found out several optimum prognostic genes for GSE39582 CRC samples by applying biomarkers related to the EMT signaling pathway (Friedman et al., 2010) and using least absolute shrinkage and selection operator (LASSO) Cox regression analysis, with the help of “glmnet” package in R. This research used linear models for microarray data (LIMMA) to conduct an analysis of DEGs between the early relapse group and the long-term survival group. We set the DEG identification threshold to *p* < 0.05 and fold change ≥ 1.2. Taking the results of the LASSO and LIMMA analyses into account simultaneously, 19 biomarkers with the best log2-fold change or λ were identified.

### Establishment of the EMT-Related Gene Signature for the Prognosis of CRC Patient

By LIMMA and LASSO Cox regression analysis, a risk score formula for the optimal prognostic EMT-related gene signature for each sample was calculated based on the relative expression of each prognostic EMT-related gene and its associated expression value, which was weighted by the LASSO Cox regression coefficient of the gene. Based on this specific risk score formula, the patients were divided into high-risk and low-risk groups by using the “Best” cutoff point (threshold) of the GSE39582 set as the cutoff point. Using the Time-dependent receiver operating characteristic (ROC) analysis, we calculate the area under the curve (AUC) for 1−, 3−, and 5-year RFS and OS and to verify the accuracy of the prognosis predicted by the signature using the ‘survivalROC’ R package in GSE39582 (Heagerty and Zheng, 2005). By Kaplan–Meier (K–M) survival curve analyses and log-rank tests, we evaluated the prognostic significance of the EMT-related signature. The distributions of patients’ risk score, survival and recurrence status were plotted to show the relationship between the calculated risk score and patient survival. In addition, with the help of the “ComplexHeatmap” R package, a heatmap was constructed with cluster analysis in view of the 19 EMT-related gene differentiation methods according to the EMT-related gene signature risk score.

### Fudan University Shanghai Cancer Center (FUSCC) Validation Cohort

To prove that the results are significant regardless of the dataset used in the study, we verified the results in the FUSCC validation cohort. This study retrospectively analyzed 104 CRC patients who underwent radical surgery at the FUSCC from 2013 to 2014. The study design was approved by the ethics committee and by the institutional review committee of our cancer center, and written informed consent was obtained from all patients. All cancer tissues were stored at −80°C. Total RNA extraction and reverse transcription were performed according to the manufacturer’s protocol. SYBR Green Supermax (Takara) was used for real-time PCR on an ABI PRISM 7500 Fast Sequence Detection System (Applied Biosystems). The primers used to amplify specific genes are shown in Supplementary Table 6. A risk score formula was constructed for patients. According to the risk score formula, the patients were divided into a low-risk group and a high-risk group by using the median risk score as the cutoff point. Kaplan–Meier analysis was used to evaluate the difference in survival between the two groups, and the log rank test was used for comparison.

### Validation of the EMT-Related Gene Signature in Independent Data Sets

To further investigate the reliability of the identified EMT-related gene signature for classification, this study verified it in four independent datasets (GSE37892, GSE33113, GSE17538, and GSE14333) using the analysis methods for each dataset. Additionally, in the TCGA dataset, besides to the expression values of each selected gene, a new risk score formula, weighted by the estimated regression coefficient in the multivariate Cox regression, was constructed for each patient. Estimation of the new EMT-related gene signature for CRC patient prognosis in the TCGA dataset was then performed.

### Correlation Between the EMT-Related-Gene Signature and Patients’ Clinicopathological Characteristics

K–M survival analyses of the indicated subtypes of various clinicopathological characteristics were performed. These characteristics included sex, age, tumor site, pathological T stage, LNM, chemotherapy, TP53 mutation status, KRAS mutation state, and BRAF mutation state. The statistical significances of the differences between them were analyzed using the t test or one-way ANOVA. The relationship between each clinicopathological characteristic and risk level was measured using the chi-square test.

### Functional Enrichment Analysis

Functional enrichment analysis of GO pathways was performed to determine significantly enriched GO terms for the genes correlated with the 19-EMT-related gene signature using the ClueGO plugin (version 2.5.6) in Cytoscape limited to biological processes (Bindea et al., 2009) and the “clusterProfiler” R package. Functional maps and clusters of enriched GO terms were obtained and visualized using a two-sided hypergeometric test with Bonferroni stepdown correction and a kappa score threshold of 0.4 and limited to level intervals 3–8 with *p* ≤ 0.05. Biological pathways for which *p* < 0.05 using functional annotation chart options with the whole human genome as background were considered significant.

### Loss-of-Function Exploration

The detailed materials and methods used in “loss-of-function exploration” can be found in the Supplementary Appendix: Methods.

### Predictive Nomograms and Clinical Usefulness

Based on the GSE39582 and TCGA cohorts, we adopted the univariable and multivariable Cox regression analyses to calculate each variable’s influence. Selecting the variables with *p* < 0.05 in the univariate model, the multivariate Cox regression algorithm was adopted. EMT-related gene signature, pathological T stage, and LNM were independent prognostic factors that could be effective in predicting RFS, and EMT-related gene signature, pathological T stage, LNM, age, and sex were independent prognostic factors that could be effective in predicting OS. Based on the results of multivariable Cox regression analysis, two nomograms that integrated clinicopathological parameters with the gene signature were formulated, applying the “rms” R package. The overall points for each patient in the GSE39582 and TCGA cohorts was calculated using these nomograms.

Decision curve analysis (DCA) integrating the risk prediction model into a clinical approach, evaluates a predictive model and visualizes the latent profit of therapy (Vickers and Elkin, 2006). Thus, DCA was performed to analyze the clinical consequences of the predictive nomogram.

### Statistical Analysis

Distribution differences between the variables examined were assessed using the χ^2^ test or Fisher’s exact test when appropriate. The ROC curves, calibration curves and DCA were used to determine the predictive accuracy of the prognostic models. The Kaplan–Meier method was used to draw survival curves, and the curves were compared using the log-rank test. Multivariate analyses were performed using the Cox proportional hazards model. All statistical analyses were performed using R (version 3.3.3^6^). All statistical tests were two-side analysis, and *p* < 0.05 was considered to indicate statistical significance.

## Results

### CRC Datasets Preparation

The detailed EMT-related gene list was collected from public databases (dbEMT: see Text Footnote 1) (Supplementary Table 1). 1,430 patients with stages I–III CRC, including 489 patients from GSE39582, 130 cases from GSE37892, 90 cases from GSE33113, 167 cases from GSE17538, 222 cases from GSE14333, and 332 cases from the TCGA database, were sampled and fully studied. Cases lacking the necessary clinicopathological or prognostic data were excluded. The detailed clinicopathological information on the five GEO datasets is displayed in Supplementary Table 2, and TCGA cohort information is shown in Supplementary Table 3. An overview of the comprehensive transcriptome analysis of an EMT-related gene signature in CRC is shown in Supplementary Figure 1.

### EMT Signaling Pathway Is Dramatically Activated in the Transition of CRC From LNM-Negative to LNM-Positive

**FIGURE 1:**
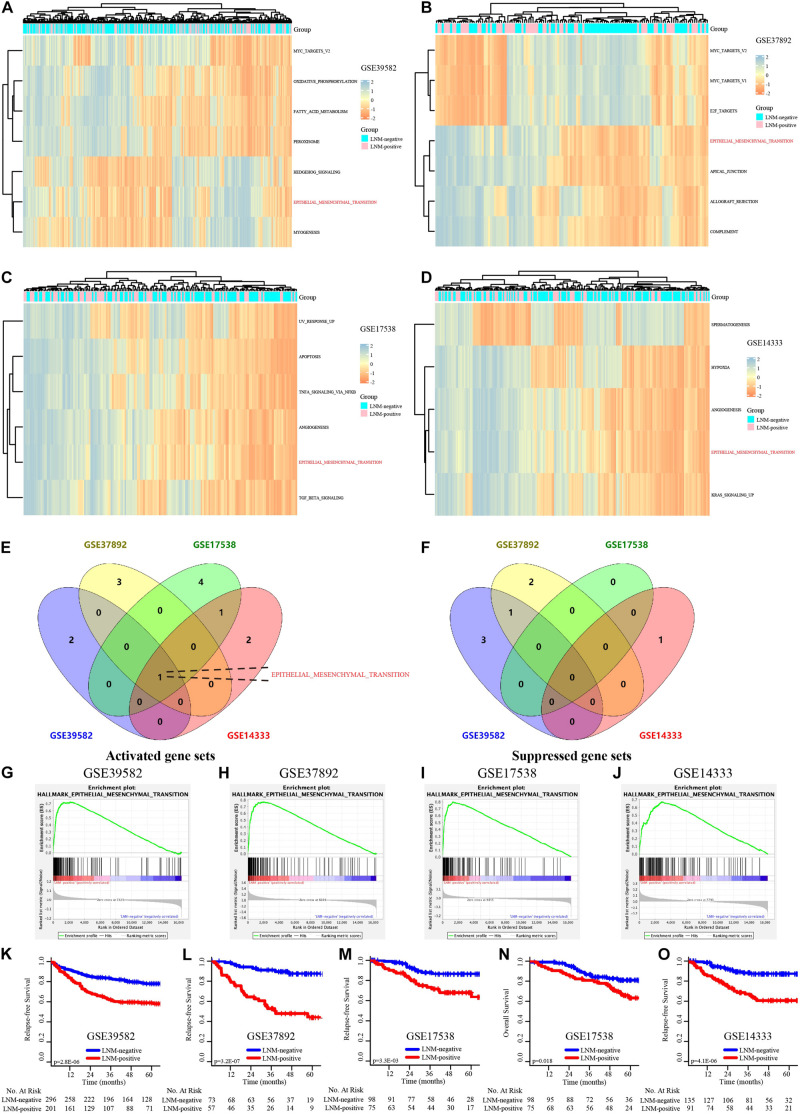
The EMT signaling pathway is dramatically activated in the CRC transition from LNM-negative to LNM-positive. **(A–D)** GSVA of the GSE39582, GSE37892, GSE17538, and GSE14333 data sets. **(E,F)** Venn diagram of the activated **(E)** and suppressed **(F)** gene sets in the indicated data sets. **(G–J)** GSEA of “HALLMARK_EPITHELIAL_MESENCHYMAL_TRANSITION” of GSE39582, GSE37892, GSE17538, and GSE14333. **(K–O)** K–M survival analysis of LNM-negative and LNM-positive CRC patients in GSE39582, GSE37892, GSE17538, and GSE14333.

We performed gene set variation analysis (GSVA) of hallmark gene sets in 4 independent GEO datasets: GSE39582, GSE37892, GSE17538, and GSE14333. The results displayed in the heatmap (Figures 1A–D) concentrated on the active EMT signaling pathway and were significantly focused between the LNM-positive and LNM-negative subtype groups (Figures 1E,F). The detailed results of the Venn plot are shown in Supplementary Table 4. GSEA of the HALLMARK_EPITHELIAL_MESENCHYMAL_TRANSITION gene set was then performed; the results indicated that the EMT signaling pathway was noticeably enriched in all datasets (Figures 1G–J). The Enrichment score (ES) is 0.73, 0.77, 0.80, and 0.67 in GSE39582, GSE37892, GSE17538, and GSE14333, respectively. Moreover, the survival outcomes for patients in the LNM-negative and LNM-positive groups were significantly different in view of the K–M analysis results (Figures 1K–O). Overall, the results of the GSVA, GSEA, and K–M survival analysis suggested that EMT-related genes might be prognostic biomarker candidates in stages I–III CRC.

### Development of the EMT-Related-Gene Signature for Prognosis

**FIGURE 2:**
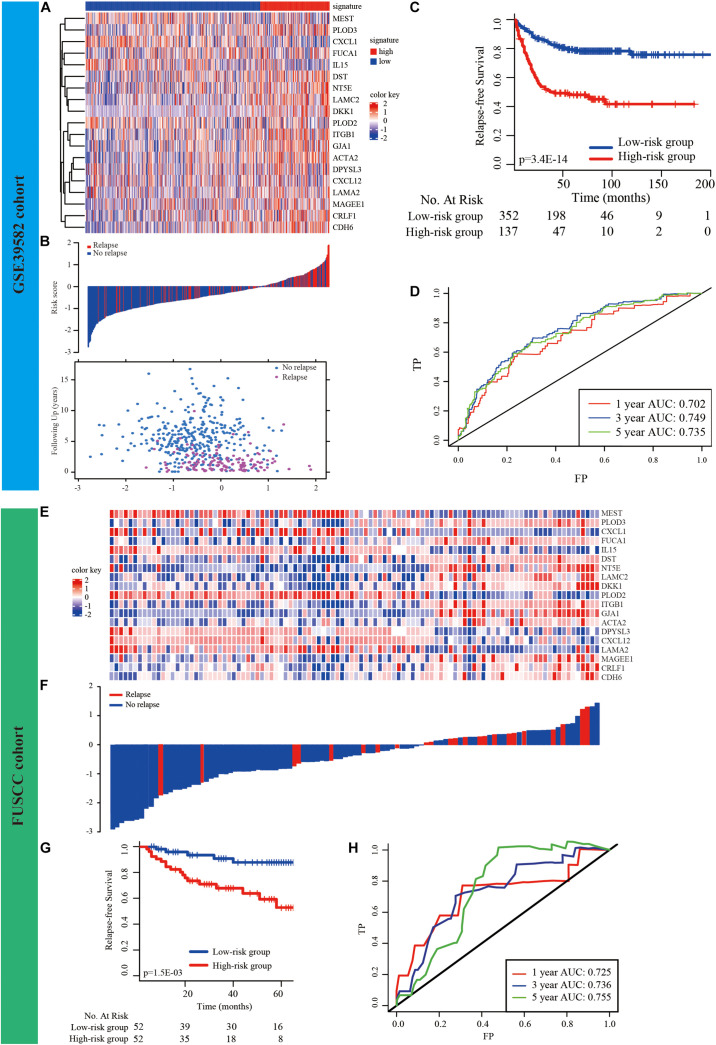
Development and validation of prognostic EMT-related gene signature. **(A)** The expression of the EMT-related genes between low− and high-risk groups was shown in the heatmap. **(B)** Relationship between the survival status/risk score rank and survival time (years)/risk score rank. **(C)** K–M survival analysis of high− and low-risk groups. **(D)** Time-dependent ROC curve for RFS. The AUC was assessed at 1−, 3−, and 5-year. **(E–H)** Validation of the prognostic EMT-related gene signature in FUSCC cohort.

LASSO and Cox regression analyses were used to screen prognosis-related EMT genes in the GSE39582 dataset. The analysis of discrepantly expressed EMT-related genes (DEEGs) between the early-relapse and long-term-survival groups in the GSE39582 dataset was performed using the LIMMA method. Thirty-one EMT-related genes were found to be related to prognosis based on LASSO analysis (Supplementary Figure 2 and Supplementary Table 5). In addition, thirteen genes (Supplementary Table 5) appeared to be differentially expressed when the LIMMA method was used; the heatmap of these genes is displayed in Supplementary Figure 3. Sifting the results of LASSO by DEEG, nineteen EMT-related genes were found to be differentially expressed between the two groups (Supplementary Figure 4). The formula used to calculate the risk score by LASSO Cox regression modeling was as follows: −0.3063694 ^∗^ (PLOD2 expression) + 0.4994757 ^∗^ (DST expression) + 0.7659238 ^∗^ (CDH6 expression) + 0.6310006 ^∗^ (ACTA2 expression) + 0.2181532 ^∗^ (CRLF1 expression) − 0.161345 ^∗^ (CXCL1 expression) − 0.2057906 ^∗^ (CXCL12 expression) + (DKK1 expression) ^∗^ 0.1861136 − (DPYSL3 expression) ^∗^ 0.3017105 + (FUCA1 expression) ^∗^ 0.4318945 + (GJA1 expression) ^∗^ 0.2273617 + (ITGB1 expression) ^∗^ 0.7700945 − (LAMA2 expression) ^∗^ 0.3587109 + (LAMC2 expression) ^∗^ 0.134651 + (MAGEE1 expression) ^∗^ 0.4941341 + (NT5E expression) ^∗^ 0.2159442 + (PLOD3 expression) ^∗^ 0.2229214 − 0.2060671 ^∗^ (MEST expression) − 0.2939619 ^∗^ (IL15 expression). CRC patients were divided into low-risk and high-risk groups according to this signature using the value at the best sensitivity and specificity as the cutoff point. The expression of EMT-related genes in the low− and high-risk groups is shown in the heatmap (Figure 2A). The distribution of relapse time and status as a result of risk score is shown in Figure 2B; it suggests that patients with lower risk scores tend to have better survival than other patients. Further K–M survival analysis indicated that the high-risk group had a notably higher relapse rate and shorter survival time than the low-risk group (*p* < 0.001, Figure 2C). Time-dependent ROC analysis at 1, 3, and 5 years after resection was conducted to determine the accuracy of the signature for predicting prognosis. The AUCs were 0.702 (95% CI, 0.695–0.722), 0.749 (95% CI, 0.721–0.768), and 0.735 (95% CI, 0.713–0.757) at survival times of 1, 3, and 5 years, respectively (Figure 2D).

### External Validation of the EMT-Related Gene Signature

First, based on our cancer center dataset (FUSCC cohort, *n* = 104, Supplementary Table 7), this novel signature could also be used for risk stratification in CRC patients, as it has substantial clinical prognostic value (*p* = 0.0015, Figures 2E–G). The AUCs were 0.725 (95% CI, 0.701–0.732), 0.736 (95% CI, 0.714–0.762), and 0.755 (95% CI, 0.730–0.767) at survival times of 1, 3, and 5 years, respectively (Figure 2H).

**FIGURE 3:**
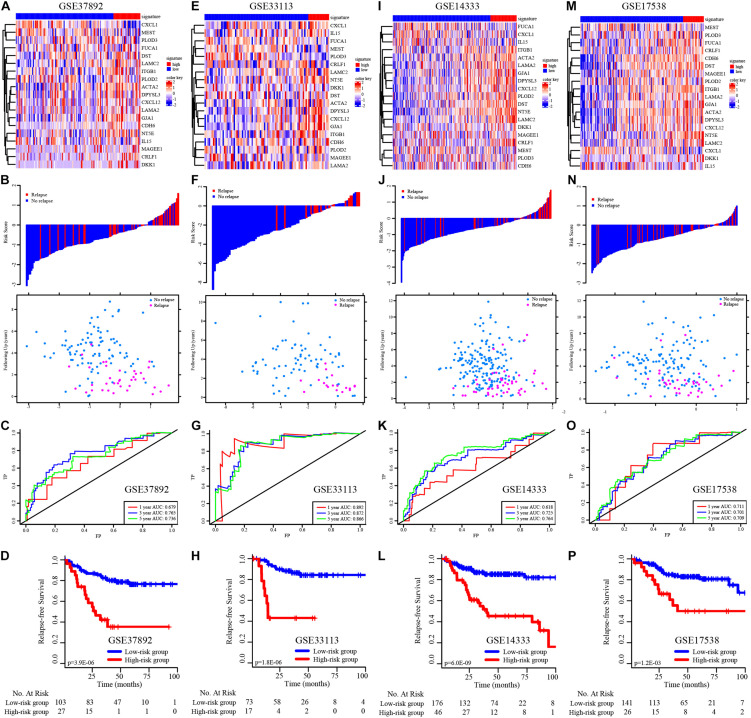
K–M survival analysis, risk score assessment by the EMT-related gene signature and time-dependent ROC curves in the external independent validation data sets. **(A–D)** The heatmap, distribution of relapse time and status related to risk scores, K–M survival analysis of high− and low-risk groups, and time-dependent ROC curve for RFS in GSE37892 set. **(E–H)** The heatmap, distribution of relapse time and status related to risk scores, K–M survival analysis of high− and low-risk groups, and time-dependent ROC curve for RFS in GSE33113 set. **(I–L)** The heatmap, distribution of relapse time and status related to risk scores, K–M survival analysis of high− and low-risk groups, and time-dependent ROC curve for RFS in GSE14333 set. **(M–P)** The heatmap, distribution of relapse time and status related to risk scores, K–M survival analysis of high− and low-risk groups, and time-dependent ROC curve for RFS in GSE17538 set.

We then validated the EMT-related gene signature based on the cases from the GEO dataset (GSE37892, GSE33113, GSE17538, and GSE14333) using the same method. The expression of EMT-related genes in the low− and high-risk groups in each dataset is shown in the heatmap (Figures 3A,E,I,M). The distribution of relapse time and status related to risk score in each dataset is shown in Figures 3B,F,J,N, respectively. The 1−, 3−, and 5-year AUCs for each dataset were considerable, indicating that the signature was accurate at predicting prognostic conditions (Figures 3C,G,K,3O). We found significantly higher survival rates in the low-risk group than in the high-risk group in each dataset (*p* < 0.001 in Figure 3D, *p* < 0.001 in Figure 3H, *p* < 0.001 in Figure 3L, and *p* = 0.001 in Figure 3P).

We subsequently validated the ability of the EMT-related gene signature to predict OS in CRC patients. The distribution of survival time and status related to risk scores in each dataset is shown in Supplementary Figures 5A,D. We observed significant prognostic values in GSE39582 (*p* < 0.001, Supplementary Figure 5C) and GSE17538 (*p* = 0.008, Supplementary Figure 5F), with 1−, 3−, and 5-year prognostic accuracies of 0.767, 0.686, and 0.682 (Supplementary Figure 5B) and 0.709, 0.612, and 0.604, respectively (Supplementary Figure 5E).

Furthermore, we used the TCGA external cohort for RFS and OS validation (Supplementary Figure 6). We found significant prognostic values in both RFS and OS prediction (*p* < 0.001, Supplementary Figures 6F,G), with 1−, 3−, and 5-year prognostic accuracies of 0.649, 0.692, and 0.702 (Supplementary Figure 6E) and 0.727, 0.764, and 0.767, respectively (Supplementary Figure 6C).

### Subgroup Analysis of the EMT-Related Gene Signature

**FIGURE 4:**
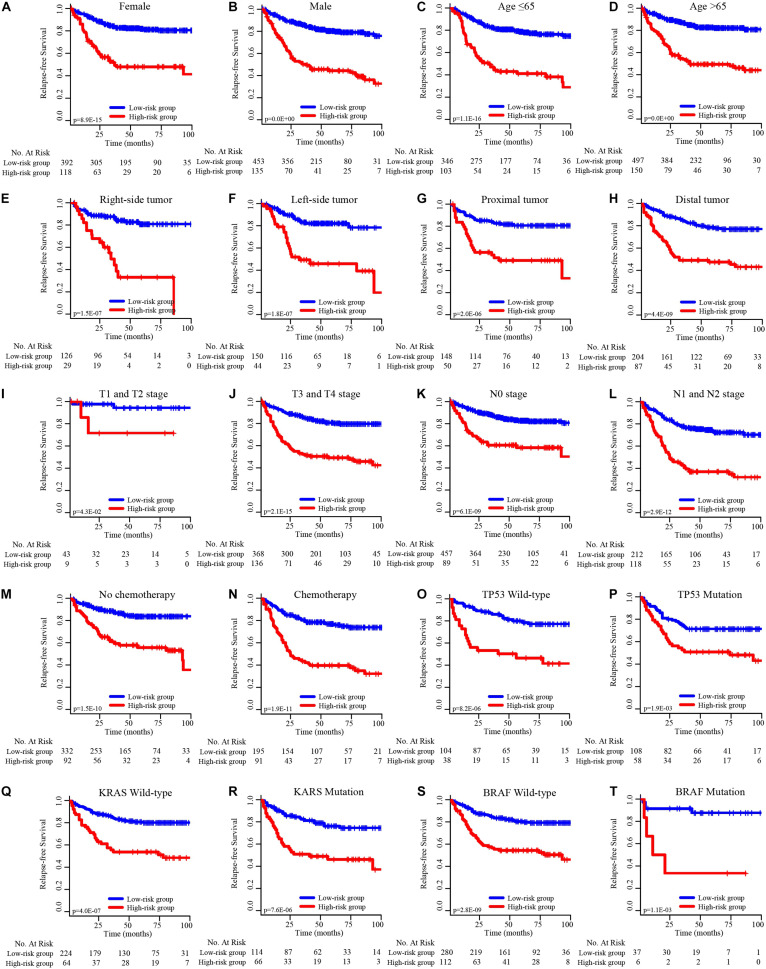
K–M survival subgroup analysis of all CRC patients according to the EMT-related gene signature stratified by clinical characteristics. **(A)** Female, **(B)** male, **(C)** age ≤ 65 years, **(D)** age > 65 years, **(E)** right-side tumor, **(F)** left-side tumor, **(G)** proximal tumor, **(H)** distal tumor, **(I)** T1 and T2 stage, **(J)** T3 and T4 stage, **(K)** N0 stage, **(L)** N1 and N2 stage, **(M)** no chemotherapy, **(N)** chemotherapy, **(O)** TP53 wild-type, **(P)** TP53 mutation, **(Q)** KRAS wild-type, **(R)** KRAS mutation, **(S)** BRAF wild-type, **(T)** BRAF mutation.

To determine the predictive power of the EMT-related gene signature in different CRC patient subgroups, the CRC patients were divided into subgroups based on individual clinical factors. Stratified analysis suggested that the 19-EMT-related signature was still a clinically and statistically significant prognostic model in patients in the different sex, age, tumor site, pathological T stage, pathological N stage, chemotherapy status, TP53 status, KRAS status, and BRAF status subgroups (Figure 4).

### Functional Implication of the EMT-Related Gene Signature

**FIGURE 5:**
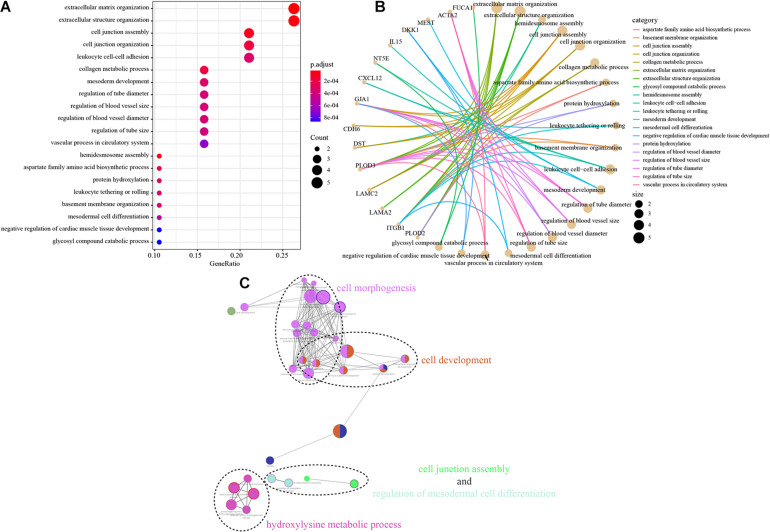
Functional analysis of the EMT-related gene signature. **(A,B)** The most significantly enriched KEGG pathways. The node size represents the number of genes in the pathways, and the color represents the pathway enrichment significance. **(C)** Functionally grouped network with enriched GO terms as nodes linked based on kappa score threshold of 0.4. Node size represents the term enrichment significance.

Next, we further explored the potential functions and signaling pathways associated with EMT-related genes in tumorigenesis, tumor invasion and metastasis. The biological function pathway analysis indicated that the EMT-related gene signature plays functional roles in 20 markedly enriched biological pathways; most of these pathways are associated with the function of promoting tumor development and metastasis, including extracellular structure organization, cell junction organization, and regulation of the blood vessel size signaling pathway (Figures 5A,B). To further classify the above pathways, Gene Ontology (GO) analysis was performed; it divided the related significantly enriched biological pathways into four functional clusters: cell morphogenesis, cell development, cell junction assembly, and hydroxylysine metabolic process (Figure 5C). The above results indicate that the identified EMT-related genes are significantly enriched in critical cancer-related biological pathways involved in CRC progression.

### Loss-of-Function Assay of Selected EMT-Related Genes Monitoring Cell Proliferation and Metastasis

We evaluated the biological roles of the selected EMT-related genes in CRC. Of the 19 EMT-related genes, only CRLF1 was significantly associated with the prognosis of CRC at both the RFS (Supplementary Figure 7) and OS (Supplementary Figure 8) levels in the TCGA cohort. Then, to investigate the role of CRLF1 in CRC, we first examined CRLF1 expression levels in CRC cell lines. Notably, we observed higher CRLF1 expression in the RKO and DLD1 cell lines and lower CRLF1 expression in the LoVo cell line (Supplementary Figures 9A,B). Furthermore, we conducted a loss-of-function assay by knocking down the expression level of CRLF1 in the RKO and DLD1 cell lines (Supplementary Figures 9C,D). CCK-8 and colony formation assays were performed to examine whether CRLF1 expression affects CRC cell proliferation and viability *in vitro*. Cell proliferation and viability were significantly decreased in RKO-si-CRLF1 and DLD1-si-CRLF1 cells compared with si-NC cells (*p* < 0.05, Supplementary Figures 9E–G). Next, we aimed to confirm the metastasis-inhibiting role of CRLF1 downregulation. As shown in Figure S9H-S9K, silencing of CRLF1 significantly inhibited migration and invasion by RKO and DLD1 cells *in vitro*. To further determine the oncogenic effects of CRLF1 in promoting CRC proliferation and metastasis *in vivo*, CRLF1 knockdown and control cells were injected into male nude mice subcutaneously and into the distal tip of the spleen, and subcutaneous tumor growth and final tumor mass were monitored. Downregulation of CRLF1 in cancer cells led to a decrease in tumor growth rate and decreased final mean tumor volume compared with control cells (Supplementary Figures 9L–N). In addition, the mice injected with RKO-si-CRLF1 cells displayed liver metastases with fewer and smaller lesions (Supplementary Figures 9O,P). Taken together, the results of the loss-of-function assay suggest that CRLF1 may play a functional role in CRC proliferation and metastasis *in vitro* and *in vivo*.

### Establishment and Evaluation of a Nomogram Incorporating the EMT-Related Gene Signature

**FIGURE 6:**
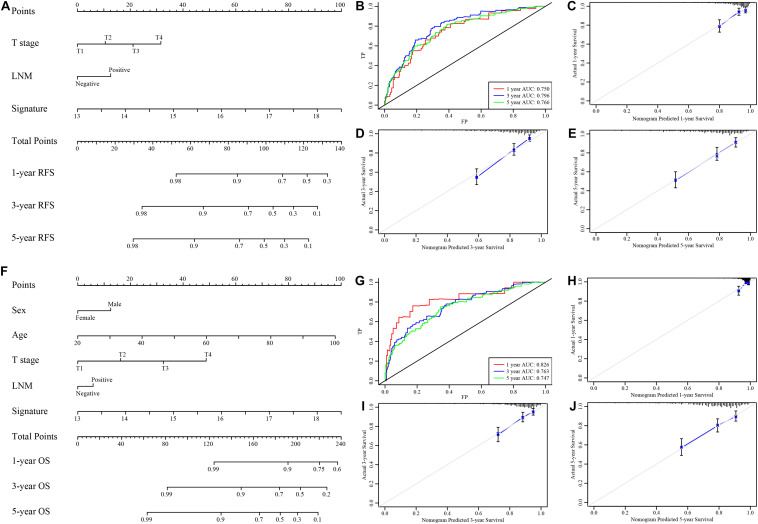
Nomograms to predict 1−, 3−, and 5-year RFS and OS in the GSE39582 set. **(A)** Nomogram for predicting the 1−, 3−, and 5-year RFS time in CRC patients. **(B)** Time-dependent ROC curve for RFS. The AUC was assessed at 1−, 3−, and 5-year. **(C–E)** Calibration curve for the prediction of 1−, 3−, and 5-year RFS. **(F)** Nomogram for predicting the 1−, 3−, and 5-year OS time in CRC patients. **(G)** Time-dependent ROC curve for OS. The AUC was assessed at 1−, 3−, and 5-year. **(H–J)** Calibration curve for the prediction of 1−, 3−, and 5-year OS.

Considering the results of univariable and multivariable analyses of RFS and OS (Supplementary Tables 8, 9), two types of nomograms incorporating the EMT-related gene signature into clinicopathological factors were established based on RFS (Figure 6A) and OS (Figure 6F) and used to quantify the possible risk of recurrence and overall prognosis for CRC patients in the GSE39582 cohort. The AUCs of the RFS nomogram and the OS nomogram were 0.750 (95% CI, 0.731–0.762), 0.796 (95% CI, 0.778–0.812), and 0.766 (95% CI, 0.745–0.787) (Figure 6B) and 0.826 (95% CI, 0.801–0.843), 0.763 (95% CI, 0.746–0.781) and 0.747 (95% CI, 0.731–0.762) (Figure 6G) at survival times of 1, 3, and 5 years, respectively. Calibration curves of the nomograms revealed no deviations from the reference line (Figures 6C–E,H–J). The results of decision curve analysis (DCA) also demonstrated that our nomogram had high potential for clinical utility (Supplementary Figure 10). To validate this result, the same protocol was applied to the TCGA cohort, and similar results were obtained (Supplementary Tables 10, 11 and Supplementary Figures 11, 12). The detailed standardized net benefits observed in the GSE39582 and TCGA cohorts are listed in Supplementary Table 12.

## Discussion

Lymph node metastasis is closely related to the postoperative recurrence of CRC and greatly affects patient survival. Clinical prognosis for stages I–III CRC often does not correspond to patients’ clinical situations due to their different genetic and epigenetic conditions (Bathe and Farshidfar, 2014). The availability of appropriate biomarkers for postoperative tumor progression would enhance the effectiveness of TNM classification and would enable physicians to formulate more individualized therapeutic and follow-up strategies.

The comprehensive transcriptome analysis conducted in the present study revealed the prognostic value of an EMT-related gene signature in CRC. Quantitative prediction results for CRC early relapse after surgical resection could be significantly improved. Applying the 19-EMT-related signature to CRC patients, obvious divergence between low-risk and high-risk patients’ recurrence and survival status was demonstrated. The signature identified in this study has been proven by several methods to be effective. The prediction results for CRC patients were highly accurate and consistent and were validated in several external independent cohorts. Furthermore, functional analysis showed that EMT-related genes are significantly enriched in cell morphogenesis, development, junctions, and critical cancer pathways that could play important roles in CRC relapse. In particular, a loss-of-function assay of selected genes indicated that EMT-related signature-associated coding genes might play functional roles in the sophisticated regulation of CRC proliferation and metastasis. By integrating the 19-EMT-related gene signature with clinicopathological factors, prognostic nomograms were constructed that can be used to predict RFS and OS probabilities at 1, 3, and 5 years after curative surgery and thereby assist clinical decisions.

The genes in the signature are strongly correlated with various types of cancer, as has been empirically proved by previous research. Of these, CXCL1, CXCL12, DKK1, FUCA1, LAMA2, ITGB1, LAMC2, NT5E, and PLOD2 have previously been reported to be strongly correlated with CRC. Wang et al. (2017) found that CXCL1 is critical for premetastatic niche formation and metastasis in CRC through its ability to recruit CXCR2-positive myeloid-derived suppressor cells. In addition, an association of high or strong expression of CXCL12 with better survival rate was observed by researchers studying CRC (Stanisavljevic et al., 2016). These conclusions about CXCL confirm the reliability of the formula established in this study. It was reported that gene expression or methylation levels of DKK1, FUCA1 and LAMA2 play a role in the occurrence, relapse and metastasis of CRC (Rawson et al., 2011; Lee et al., 2012; Ezawa et al., 2016; Rui et al., 2019). DKK1 is considered the upstream gene of the extracellular inhibitor of Wnt signaling. Thus, the amount of DKK1 methylation is closely related to prognosis status in CRC. It has also been observed that methylation of LAMA2 at the CpG site in CRC tumors was different compared to that in matched tumor-adjacent normal tissues. Thus, LAMA2 is hypermethylated in CRC and may serve as a potential biomarker of the tumor process. In patients with stage III CRC, 5-year RFS rates were significantly higher in an ITGB1-underexpression group than in an ITGB1-overexpression group, indicating that under expression of ITGB1is a risk factor for tumor recurrence, as was found in this study. Some scientists have demonstrated that ITGB1 alters the expression of N-cadherin, E-cadherin, and vimentin, proteins that are related to invasion and migration, thereby promoting invasive cell proliferation, motility, and metastasis (Ha et al., 2019). Huang et al. (2017) investigated the role of LAMC2 in CRC and suggested that overexpression of LAMC2, which was related to higher TNM stage and worse survival, promotes proliferation, migration, and invasion in CRC. Moreover, it was reported that patients with high serum NT5E expression had higher TNM stage and shorter OS than those with low NT5E expression. Du et al. (2019) demonstrated that PLOD2, which acts in the STAT3 signaling pathway, can inhibit the expression of HK2, leading to decreased cell proliferation, invasion and aerobic glycolysis in CRC cells and that higher PLOD2 expression correlated with longer OS. These findings further corroborate the signature established in this study.

The expression of CDH6, DPYSLE3, GJA1, IL15, and ACTA2 is also relevant to survival in many types of cancers, but its mechanism of action in CRC requires further study to explore its impact (Looyenga et al., 2013; Ji et al., 2018; Sun et al., 2018; Baek et al., 2019). Overexpression of CDH6 is closely associated with short OS in osteosarcoma patients. CDH6 expression was also found to be highly related to p53 expression in high-grade serous ovarian cancer (Ji et al., 2018). It has been reported that DPYSLE3 can reduce the expression of the EMT regulators SNAIL and TWIST and regulate cell proliferation in the CLOW subset of triple-negative breast cancers (Matsunuma et al., 2018). It was shown that GJA1 helps maintain cell differentiation and prevent transformation in endometrial carcinoma (Falck and Klinga-Levan, 2013). The expression of IL15 is a type of ramification in gastric cancer mesenchymal stem cells. Previous research demonstrated that IL15 can trigger the further expression of EMT and promote GC cell migration (Sun et al., 2018). It was also demonstrated that induction of ACTA2 by EGFR and HER2 dimerization is regulated through a JAK2/STAT1 signaling pathway and that abnormal ACTA2 expression accelerates the invasiveness and metastasis of breast cancer cells (Jeon et al., 2017).

In our research, we also identified certain genes, such as DST and CRLF1, that are worthy of further research. DST was reported to act as a tumor-specific alternative splicing factor in head and neck squamous cell carcinoma (Li et al., 2014). However, its relationship with cancers such as CRC needs further investigation. In view of the fact that high expression of CRLF1 is significantly related to poor prognosis, we evaluated the biological roles of CRLF1 in CRC. CRLF1 was significantly overexpressed in CRC cells. Remarkably, knockdown of CRLF1 expression significantly suppressed cell proliferation and migration capacity in the cells studied *in vitro*. Furthermore, downregulation of CRLF1 significantly inhibited the proliferation and metastasis of CRC cells *in vivo*. The loss-of-function assay indicated that CRLF1 might play functional roles in the sophisticated regulation of colon cancer progression, suggesting that it could be a potential therapeutic target for CRC.

This study indeed has some limitations. Firstly, it is based on data obtained from public datasets and does not include prospective testing in clinical trials. Besides, the underlying mechanism of action of the 19 identified EMT-related genes in the relapse of CRC will require further research.

## Conclusion

We developed a 19-EMT-related mRNA signature that can be used to effectively sort CRC patients into low− and high-risk groups for postoperative relapse. Further functional analysis and loss-of-function assays showed that the identified EMT-related genes were significantly enriched in critical cancer pathways that could play important roles in CRC proliferation, metastasis and recurrence. Nomograms based on the EMT-related signature were developed and validated to assist physicians with clinical decision-making. Together, this comprehensive transcriptomic analysis highlights a functional role for the EMT-related gene signature and uncovers a potential prognostic and therapeutic biomarker for CRC.

## Data Availability Statement

The datasets generated for this study can be found in online repositories. The names of the repository/repositories and accession number(s) can be found in the article/Supplementary Material.

## Ethics Statement

The studies involving human participants were reviewed and approved by The Ethical Committee and Institutional Review Board of the Fudan University Shanghai Cancer Center. The patients/participants provided their written informed consent to participate in this study.

## Author Contributions

SM, WD, and ZZ had the idea for this study and undertook the statistical analysis. WX and LH supervised the acquisition of the data. QL, GC, RW, YL, and LZ provided statistical advice. All authors contributed to interpretation of the results. SM, ZZ, and RG wrote the article. QL, LG, and SC revised the article and other authors contributed to the content. All authors approved the final version of the manuscript, including the authorship list.

## Conflict of Interest

The authors declare that the research was conducted in the absence of any commercial or financial relationships that could be construed as a potential conflict of interest.

## Footnotes

1 http://dbemt.bioinfo-minzhao.org/

2 http://software.broadinstitute.org/gsea/msigdb/index.jsp

3 http://software.broadinstitute.org/gsea/index.jsp

4 http://www.ncbi.nlm.nih.gov/geo/

5 https://tcga.xenahubs.net

6 www.r-project.org

## Abbreviations

CRC: colorectal cancer
LNM: lymph node metastasis
FUSCC: Fudan University Shanghai Cancer Center
EMT: epithelial mesenchymal transformation
MET: mesenchymal-epithelial transition
TNM: tumor-node-metastasis
GEO: gene expression omnibus
TCGA: the Cancer Genome Atlas
DCA: decision curve analysis
GSVA: gene set variation analysis
GSEA: gene set enrichment analysis
AUC: the area under the curve
ROC: receiver operating characteristic
LIMMA: linear models for microarray data
DEGs: differentially expressed genes
LASSO: least absolute shrink-age and selection operator
RFS: relapse-free survival
OS: overall survival
K–M: Kaplan–Meier
DEEG: discrepantly expressed EMT-related genes
HR: hazard ratio
CI: confidence interval
SD: standard deviation.
