# Supplementary material for: Comprehensive Transcriptomic Analysis Reveals Prognostic Value of an EMT-Related Gene Signature in Colorectal Cancer
Source: Front Cell Dev Biol. 2021 Jun 15;9:681431. doi: 10.3389/fcell.2021.681431 (PMC8239228; doi:10.3389/fcell.2021.681431)
Supplement: Supplementary file 1 [file Data_Sheet_1.docx]

**Supplemental Information**

**Supplementary Appendix:** Methods.

***Cell culture***

The human colon cancer cell lines (RKO, LoVo, DLD1, HCT116, and SW620 (Type Culture Collection Cell Bank, Chinese Academy of Sciences)) used for cell experiments were cultured in medium supplemented with 10% FBS (Gibco, Life Technology, Austria), and 1% antibiotics and maintained at 37°C in a humidified atmosphere containing 5% CO2 in a cell incubator (Thermo Fisher Scientific).

***Antibodies and reagents***

The following antibodies and reagents were used: anti-GAPDH antibody (10494-1-AP, Proteintech), anti-CRLF1 antibody (DF8930, Affinity Biosciences). HRP-conjugated secondary antibodies goat anti-rabbit IgG (H+L) (SA00001-2) were purchased from Proteintech Group (Rosemont, IL, USA).

***Quantitative real-time PCR***

Total RNA was isolated by using TRIzol reagent (10296010, Thermo Fisher Scientific (Waltham, MA, USA)), according to the manufacturer’s instructions. SYBR Green Supermix (Takara) was used to perform real-time polymerase chain reaction (PCR) on a QuantStudio™ 7 Flex Real-Time PCR System platform (Thermo Fisher Scientific (Waltham, MA, USA)) following standard protocols. The expression of β-actin was set as an endogenous control, and the expression of genes was calibrated to that of the corresponding control cells. Data analyses were conducted by QuantStudio Real-Time PCR Software. RQ values (relative quantified value of mRNA expression) were calculated by the same software and analyzed by two-tailed Student’s t-test to determine significant differences between two groups. All primers designed for qRT-PCR are as follows:

CRLF1 sense 5’-CCCAGAGAAACCCGTCAACAT-3’;

anti-sense 5’-ACTGTGTGGTACTCCTCACAT-3’;

β-actin sense 5’-CGCGAGAAGATGACCCAGAT-3’;

anti-sense 5’-GGGCATACCCCTCGTAGATG-3’

***Western blotting***

RIPA lysis buffer was used for protein extraction. Protein was separated by SDS-PAGE (6–20% gel) and then transferred to PVDF membranes. Western blotting was performed using primary antibodies against CRLF1 (DF8930, 1:500, Affinity Biosciences), GAPDH (10494-1-AP, 1:5000, Proteintech), and a secondary antibody (anti-rabbit IgG, 1:7500, Cell Signaling Technology). Immunoreactive proteins were detected by ECL (Pierce, Thermo Scientific) using a Bioimaging System after incubation with species-specific horseradish peroxidase-conjugated secondary antibodies. GAPDH served as the loading control.

***RNA interference***

Effective siRNA oligonucleotides that targeting CRLF1 were purchased from Guangzhou Ribobio Company (Guangzhou, China) and were transfected using Lipofectamine RNAiMax (Invitrogen) according to the manufacturer’s instructions. The siRNA sequences used were as follows:

siRNA 1# of CRLF1 sense 5’-GGCUCUCUUACGCCCUAU dTdT-3’;

anti-sense 5’-AUAGGGCGUAAAGAGAGCC dTdT-3’;

siRNA 2# of CRLF1 sense 5’-CACGCUGGAUAUCCUGGAU dTdT-3’;

anti-sense 5’-GUGCGACCUAUAGGACCUA dTdT-3’.

The expression levels of CRLF1 were verified by qRT-PCR and western blotting assays.

***Cell viability and colony formation assays***

Cell viability: A count of 2 × 10^3^ cells/well was plated into 96-well plates in triplicate and were allowed to adhere overnight. After adherence, 10 μl/100 μl of CCK8 solution was added to each well to co-incubate for 2 h at 37°C. Then, cell viability was measured by spectrophotometrically at 450 nm, and this was recorded as day 1. After 2, 3, 4, 5, 6 and 7 days, cell viability and proliferation were re-assessed.

For the colony formation assays, 400 cells in 2 mL of medium per well were seeded into 6-well plates and cultured for 10-14 days. The colonies were fixed with methanol for 10 min and stained with 0.5% crystal violet for 15 min. Each experiment was performed in three times.

***Transwell migration and invasion assays***

For the cell migration assay, cells were harvested, washed twice with PBS, resuspended in DMEM without FBS and counted by a Vi-CELL XR counter (Beckman Coulter). Transwell (8 μm, 353097, Corning) chambers were inserted into the corresponding wells in a 24-well plate that already contained 500 μL DMEM with 10% FBS. Cells at a density of 2 × 10^4^ cells/well were placed into the upper chamber and incubated for 48 h. For the cell invasion assay, cells at a density of 5 × 10^4^ cells/well were placed in serum-free medium in the upper well of 24-well Transwell inserts coated with Matrigel and incubated for 48 h. For both the cell migration assay and invasion assay, following incubation, the medium of the upper chambers was discarded, and migrated cells on the lower side were fixed and stained with 4% paraformaldehyde at room temperature for 30 min, stained with 0.5% crystal violet at room temperature for 30 min, and washed with PBS 3 times. Cells were observed, and images were captured with an Olympus microscope system and then counted in three out of eight randomly chosen, equally divided areas, followed by two-tailed Student’s t-test to determine significant differences between two groups.

***Xenotransplant murine models and metastasis assay in vivo***

CRC cells (5 × 10^6^ cells/mouse) were suspended in 100 μL Matrigel, injected subcutaneously into the right flank of nude mice (n = 6, male; 5-week-old Balb/C athymic nude mouse), and allowed to grow for 4 weeks. Tumor growth was monitored using calipers every 4 days. Four weeks after tumor cell injection, animals were euthanized when tumors reached approximately 10% of body weight. Primary tumors and organs were harvested and fixed in 10% formalin and paraffin embedded for pathological analysis. For the metastasis assay, RKO-si-CRLF1 or vector cells (1 × 10^6^ cells) were implanted into the spleens of nude mice and allowed to grow for 6 weeks. All animal studies were conducted in accordance with the animal care guidelines at FUSCC.

***Statistical analysis***

Data from in vitro experiments are presented as the mean ± SD, and the difference was analyzed using one-way ANOVA or Student’s t-test. All statistical analyses were performed using SPSS software (version 22.0; SPSS, Chicago, IL) and GraphPad Prism 6 (La Jolla, CA, USA). All confidence intervals (CIs) were stated at the 95% confidence level. P values <0.05 were considered statistically significant.


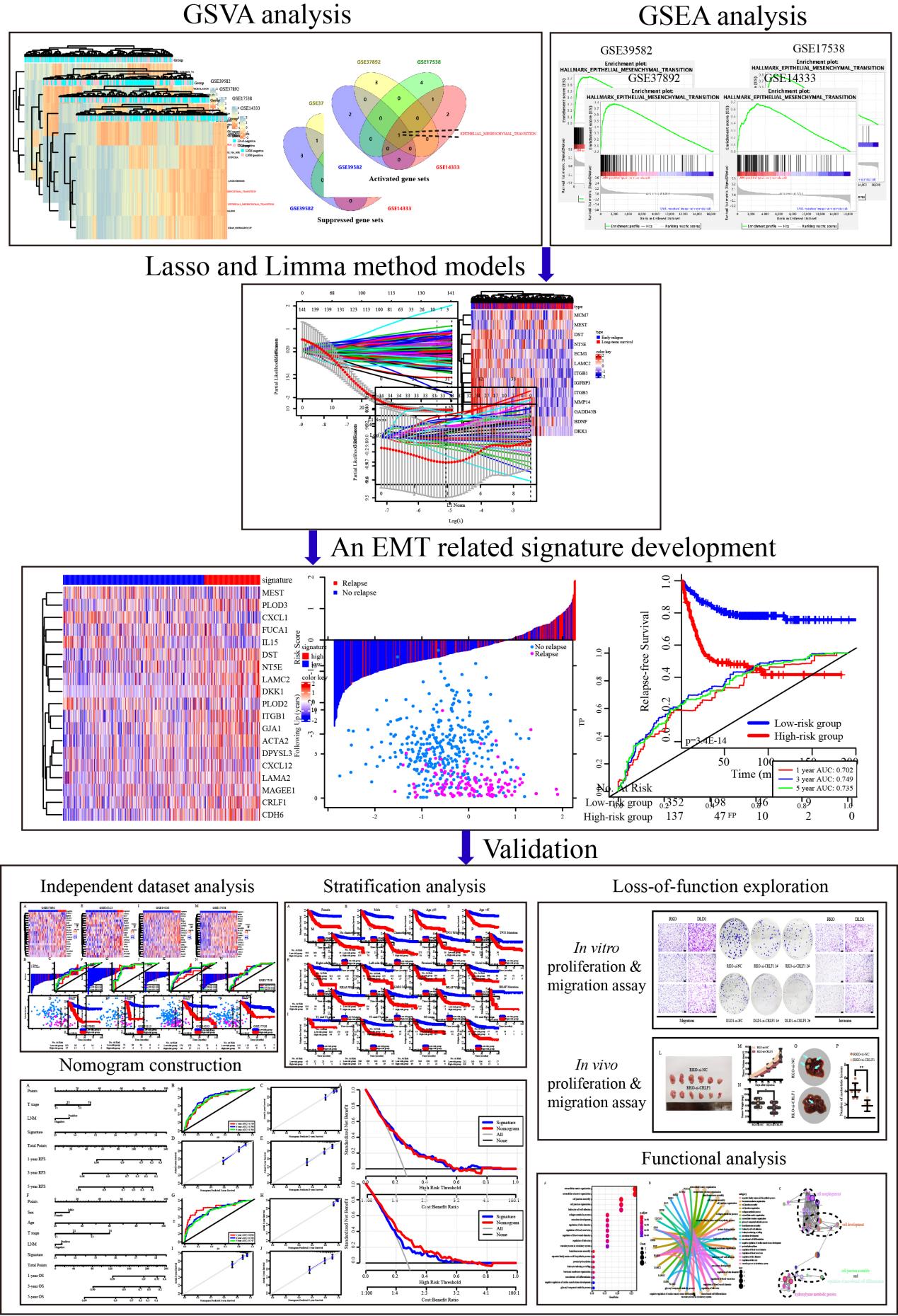


**Figure S1. Study overview.**

The EMT signaling pathway was the only common enriched pathway in the CRC transition from LNM-negative to LNM-positive by GSVA and GSEA analyses. Then, an EMT-related gene signature was identified using LASSO and LIMMA methods. Independent datasets analysis and stratification analysis showed that the EMT-related gene signature was reliable in distinguishing high- and low-risk CRC patients. Functional analysis and loss-of-function exploration in vitro and in vivo indicated that the EMT-related-signature-associated coding genes might play functional roles in the sophisticated regulation of CRC proliferation and metastasis. Eventually, nomograms integrating the EMT-related gene signature and clinicopathological risk factors were constructed as numerical prediction tools to assess clinical prognosis and to assist clinical decision.


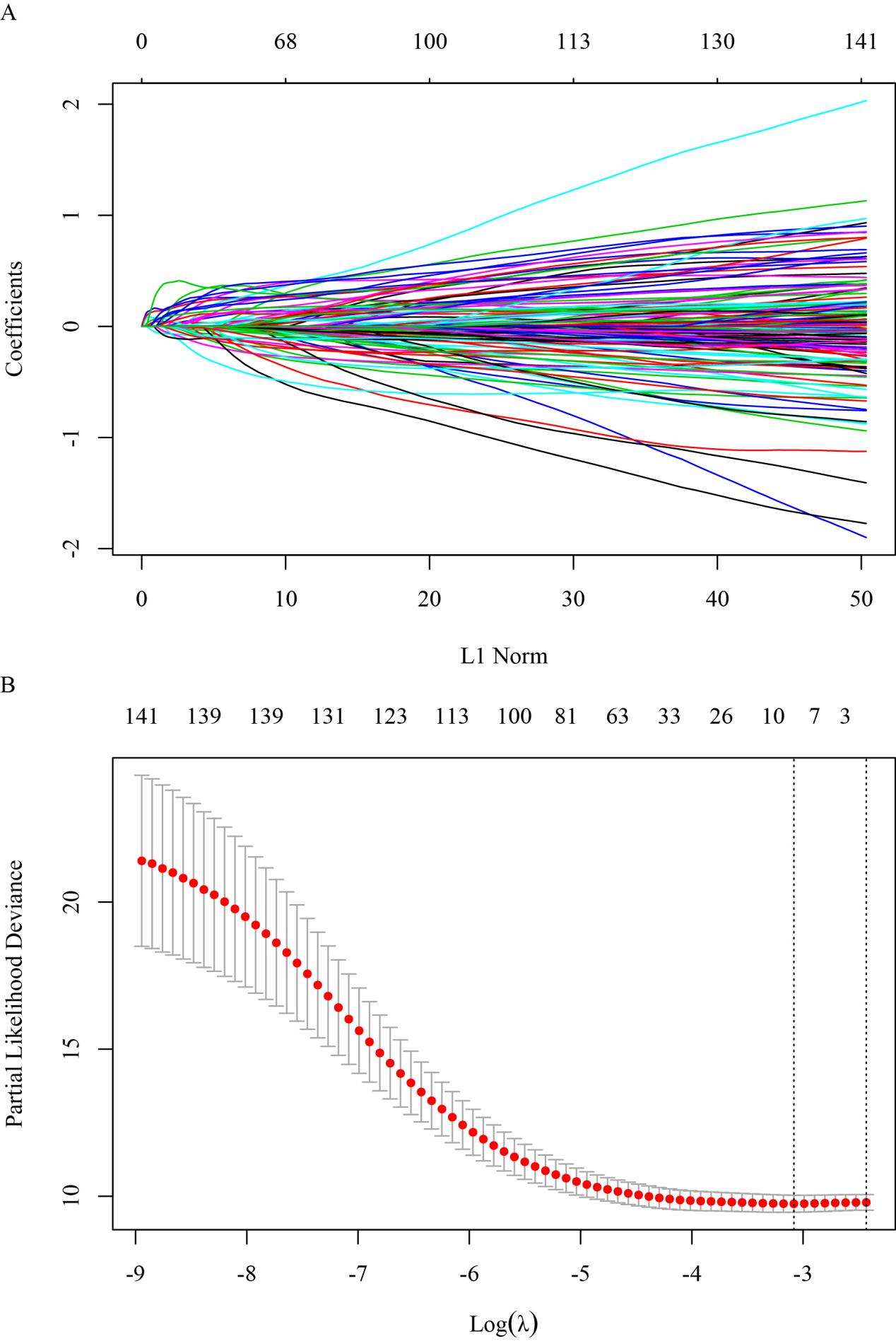


**Figure S2:** LASSO coefficient profiles of the early relapse-associated EMT-related genes.

**
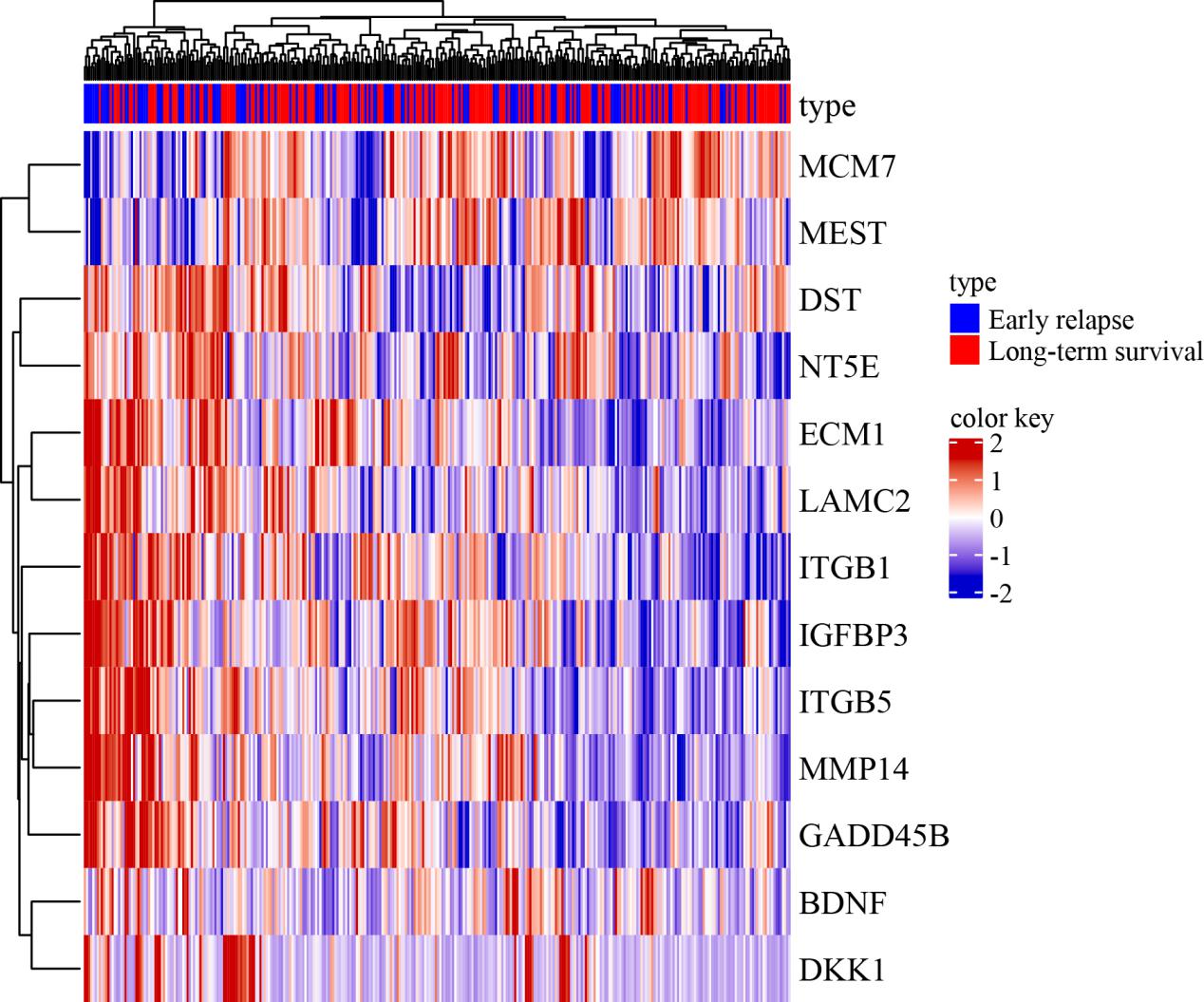
**

**Figure S3:** The heatmap of 13 genes found differentially expressed using LIMMA method.

**
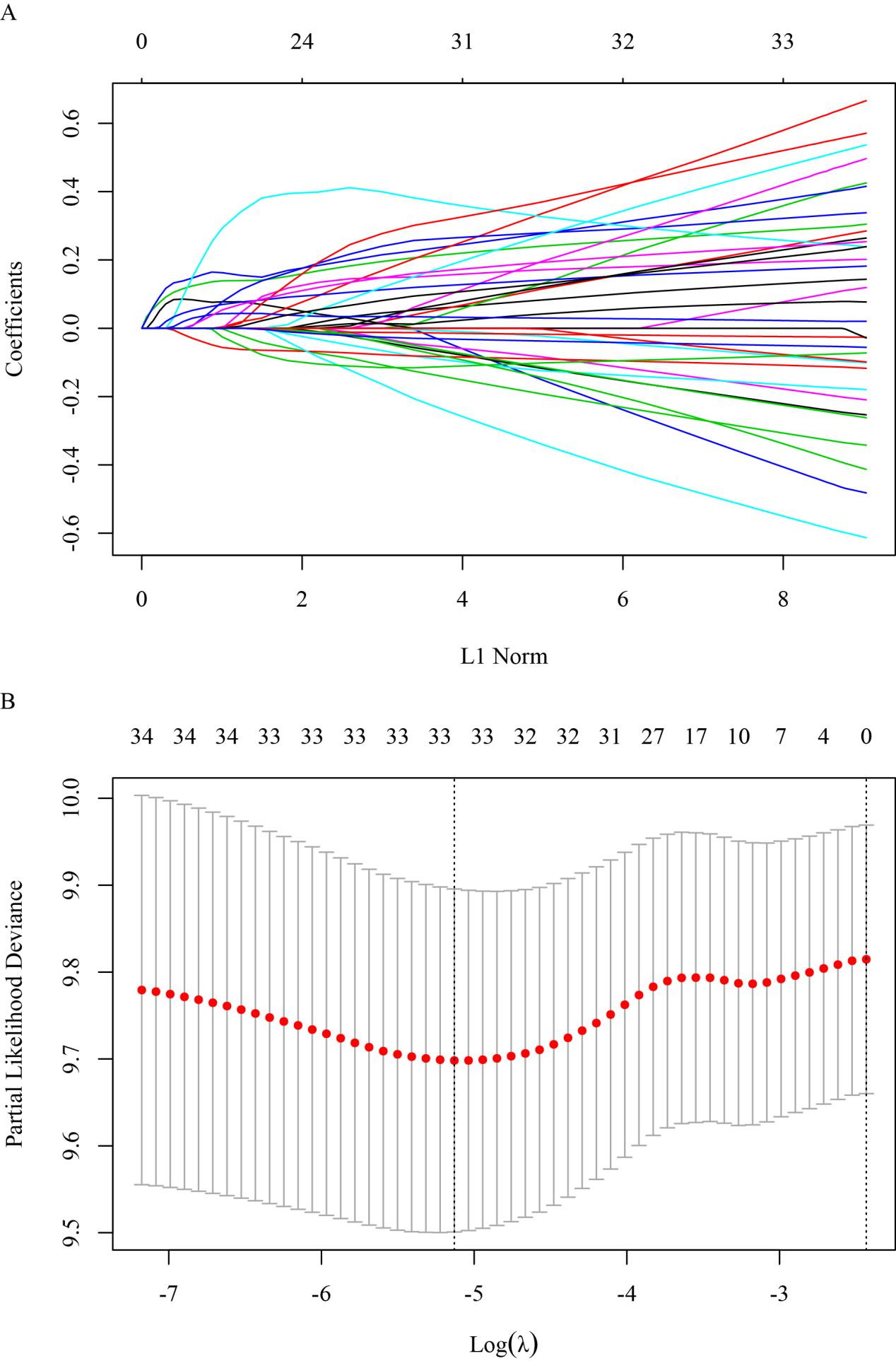
**

**Figure S4:** LASSO Cox regression after sifting the results of LASSO by discrepantly expressed EMT-related genes (DEEG).

**
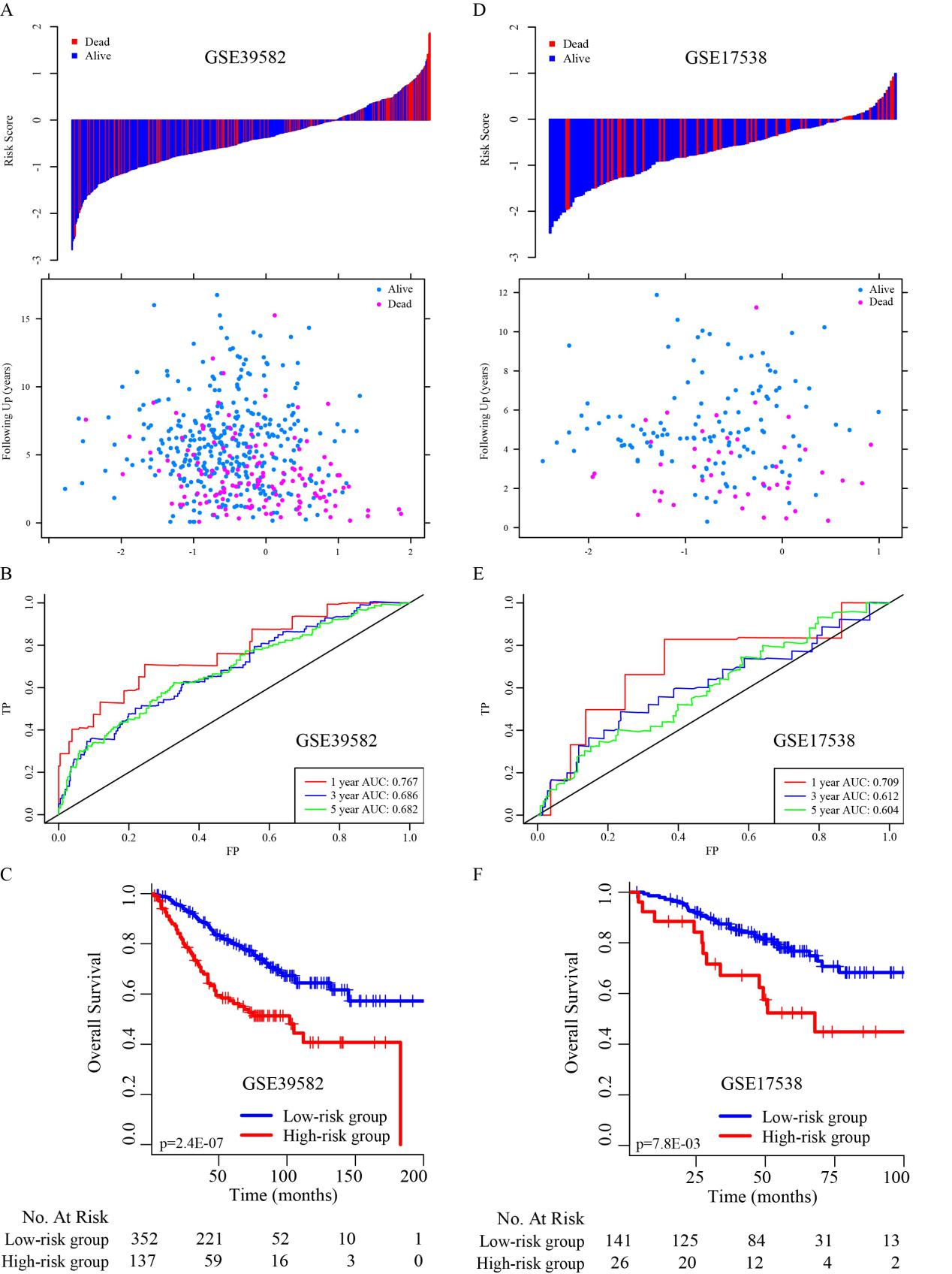
**

**Figure S5:** Validation of the 19-EMT-related signature for OS prediction in the GSE39582 and GSE17538 cohorts. The expression pattern of 19-EMT-related signature and the distribution of survival status of CRC patients in the GSE39582 (A) and GSE17538 (D) cohorts. Time dependent ROC curves at 1, 3, 5-year OS in the GSE39582 (B) and GSE17538 (E) cohorts. Kaplan-Meier survival curves of OS between high-risk and low-risk patients in in the GSE39582 (C) and GSE17538 (F) cohorts.

**
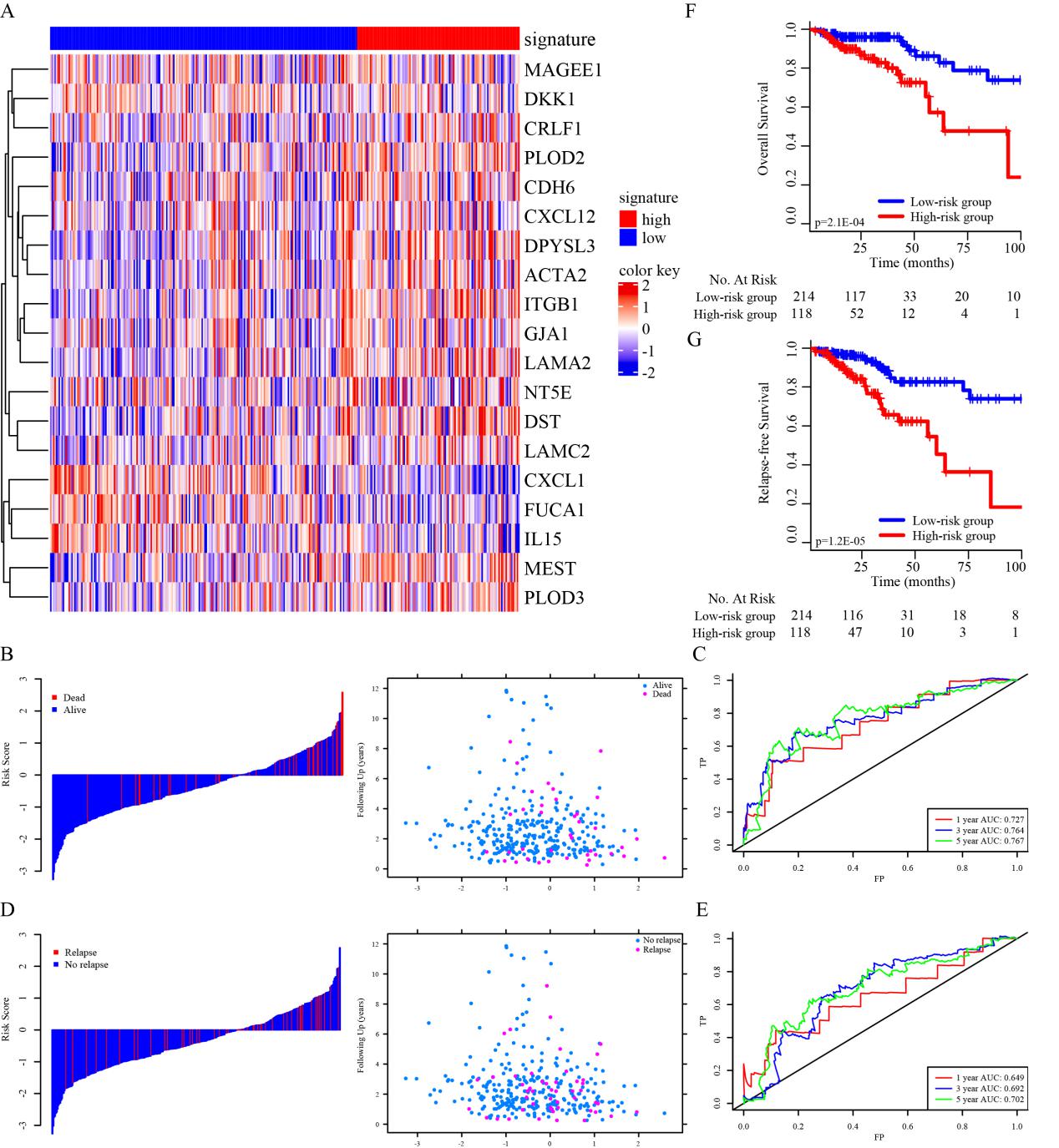
**

**Figure S6:** Validation of the 19-EMT-related signature in TCGA cohort. (A) The expression pattern of 19-EMT-related signature. (B) The distribution of survival status of CRC patients. (C) Time dependent ROC curves at 1, 3, 5-year OS. (D) The distribution of relapse status of CRC patients. (E) Time dependent ROC curves at 1, 3, 5-year RFS. (F) Kaplan-Meier survival curves of OS between high-risk and low-risk patients in TCGA cohort. (G) Kaplan-Meier survival curves of RFS between high-risk and low-risk patients in TCGA cohort.

**
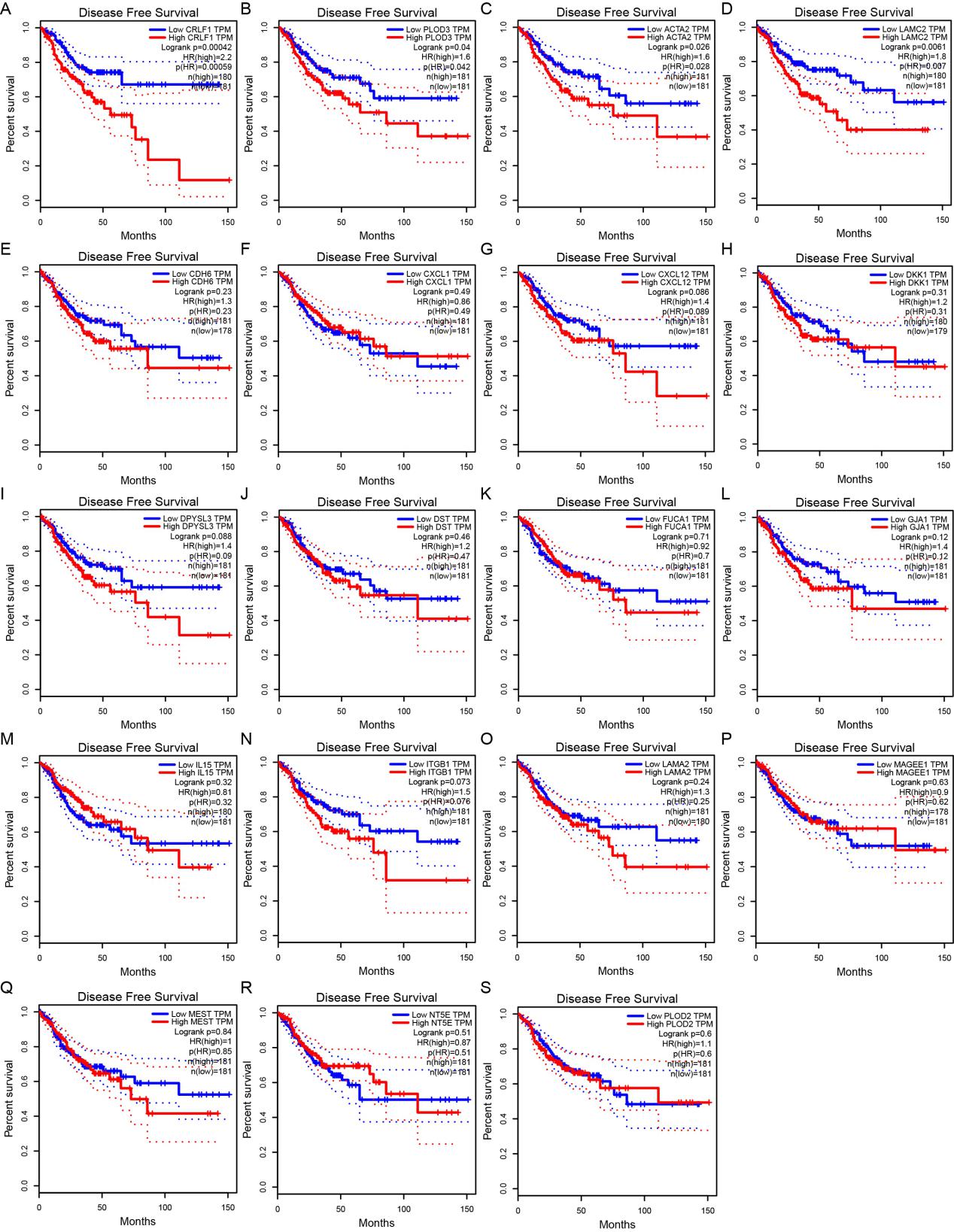
**

**Figure S7:** Kaplan-Meier survival curves of relapse-free survival between high and low expression of 19 EMT-related genes in TCGA cohort.

**
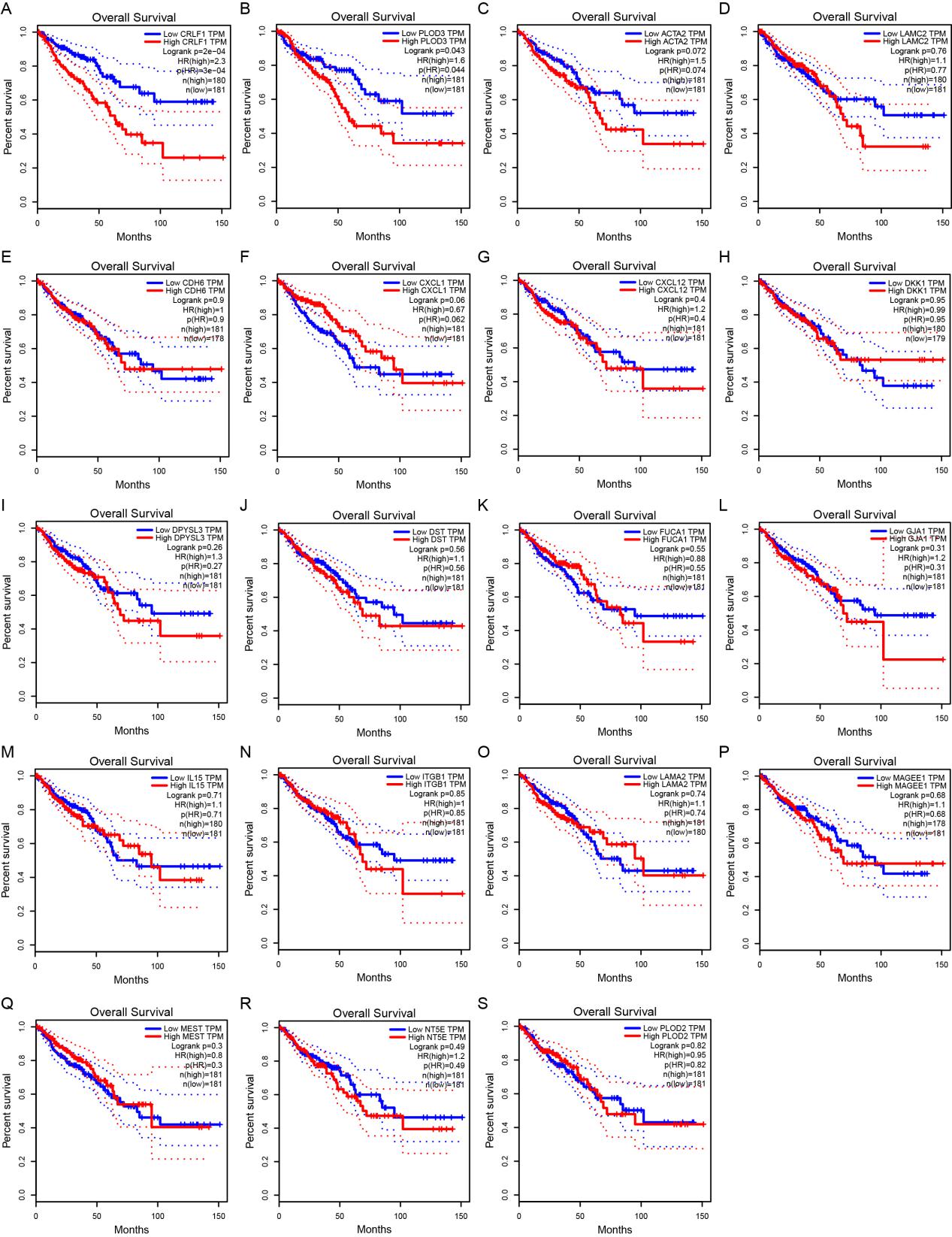
**

**Figure S8:** Kaplan-Meier survival curves of overall survival between high and low expression of 19 EMT-related genes in TCGA cohort.


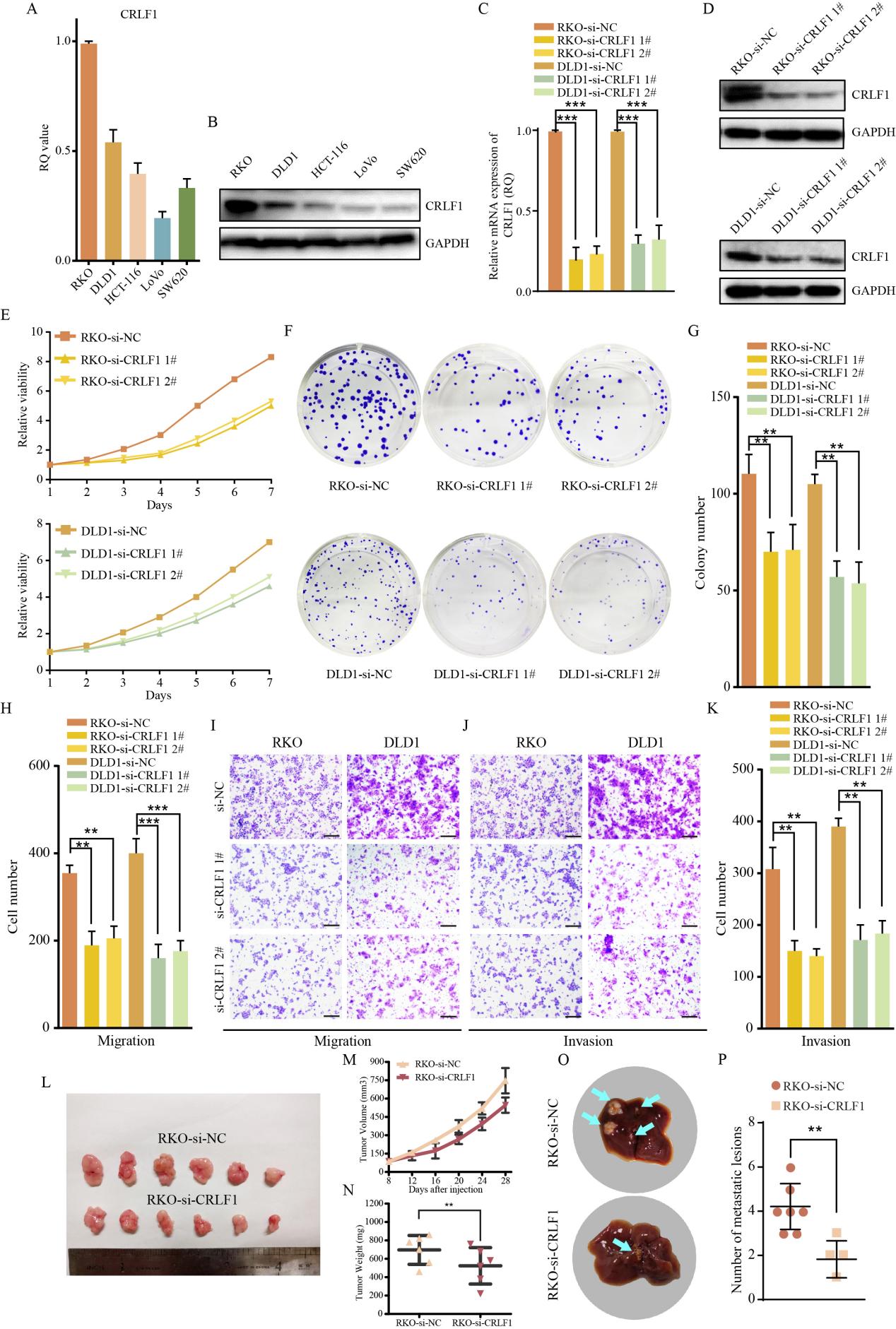


**Figure S9. Loss-of-function assay of CRLF1 regulating cell proliferation and metastasis.**

(A-B) Expression levels of CRLF1 in CRC cell lines using qRT-PCR analysis and Western blotting

(C-D) Knockdown of CRLF1 mRNA and protein with two different siRNAs (si-CRLF1 1# and 2#) in RKO and DLD1 cells was evidenced by qRT-PCR analysis and western blotting assays, respectively. β-Actin was used for normalization for the qRT-PC assays, and GAPDH was used as a loading control for the western blotting assays. The data are presented as the mean ± SD.

(E) CRLF1 knockdown significantly inhibited cell viability. The data are presented as the mean ± SD.

(F-G) CRLF1 knockdown inhibited the colony formation ability of CRC cells.

(H-I) Migration assays were used to determine the effects of CRLF1-depleted on the migration ability of CRC cells. **p <0.01, ***p <0.001, Student’s t-test, mean ± SD, scale bar: 100 µm.

(J-K) Invasion assays were used to determine the effects of CRLF1-depleted on the invasion ability of CRC cells. **p <0.01, Student’s t-test, mean ± SD, scale bar: 100 µm.

(L) Representative photographs of tumor at the end of study.

(M-N) Tumors volumes and tumor weight were measured on the indicated days. Data were expressed as means ± SD in three independent experiments. **p<0.01.

(O) The gross images of liver metastases observed in the nude mice injected with RKO cells. Arrows represent metastatic tumors.

(P) The number of metastatic lesions was smaller in the RKO-si-CRLF1 group than in the control group. Data were expressed as means ± SD in three independent experiments. **p<0.01.

**
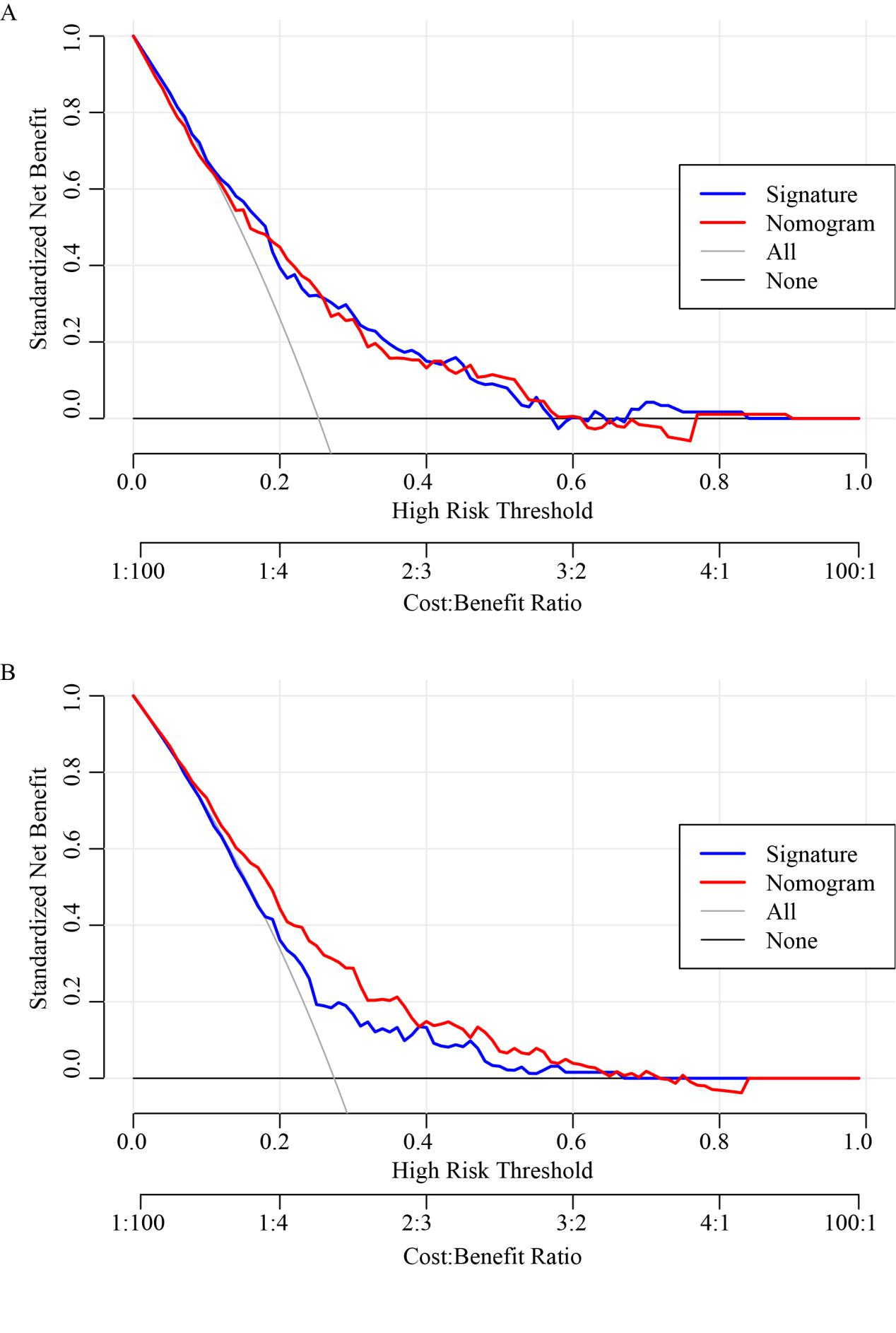
**

**Figure S10:** (A) Decision curve analysis of the nomogram and signature for the relapse-free survival prediction of stage I-III CRC patients in the GSE39582 cohort. (B) Decision curve analysis of the nomogram and signature for the overall survival prediction of stage I-III CRC patients in the GSE39582 cohort.

**
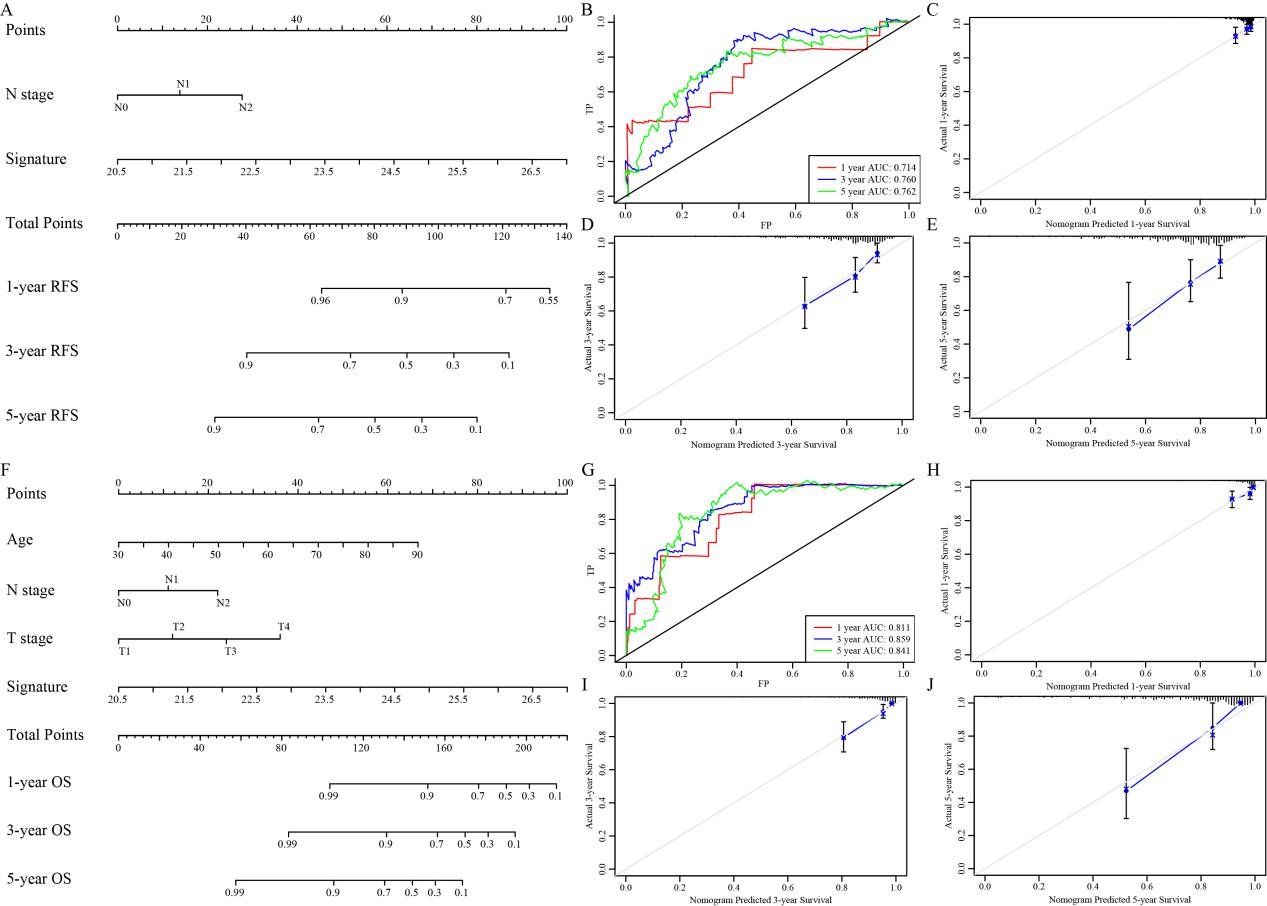
**

**Figure S11:** (A) Nomograms convey the results of prognostic models combining the 19-EMT-related signature and pathological N stage to predict RFS of patients with CRC in TCGA cohort. (B) The AUCs at 1-, 3-, and 5-year RFS prediction were 0.714, 0.760, and 0.762, respectively. The x-axis is nomogram-predicted probability of survival and y-axis is actual survival. The reference line is 45^◦^ and indicates perfect calibration [C (1-year), D (3-year), E (5-year)]. (F) Nomograms convey the results of prognostic models combining the 19-EMT-related signature and three clinicopathological characteristics to predict OS of patients with CRC in TCGA cohort. (G) The AUCs at 1-, 3-, and 5-year OS prediction were 0.811, 0.859, and 0.841, respectively. The x-axis is nomogram-predicted probability of survival and y-axis is actual survival. The reference line is 45^◦^ and indicates perfect calibration [H (1-year), I (3-year), J (5-year)].

**
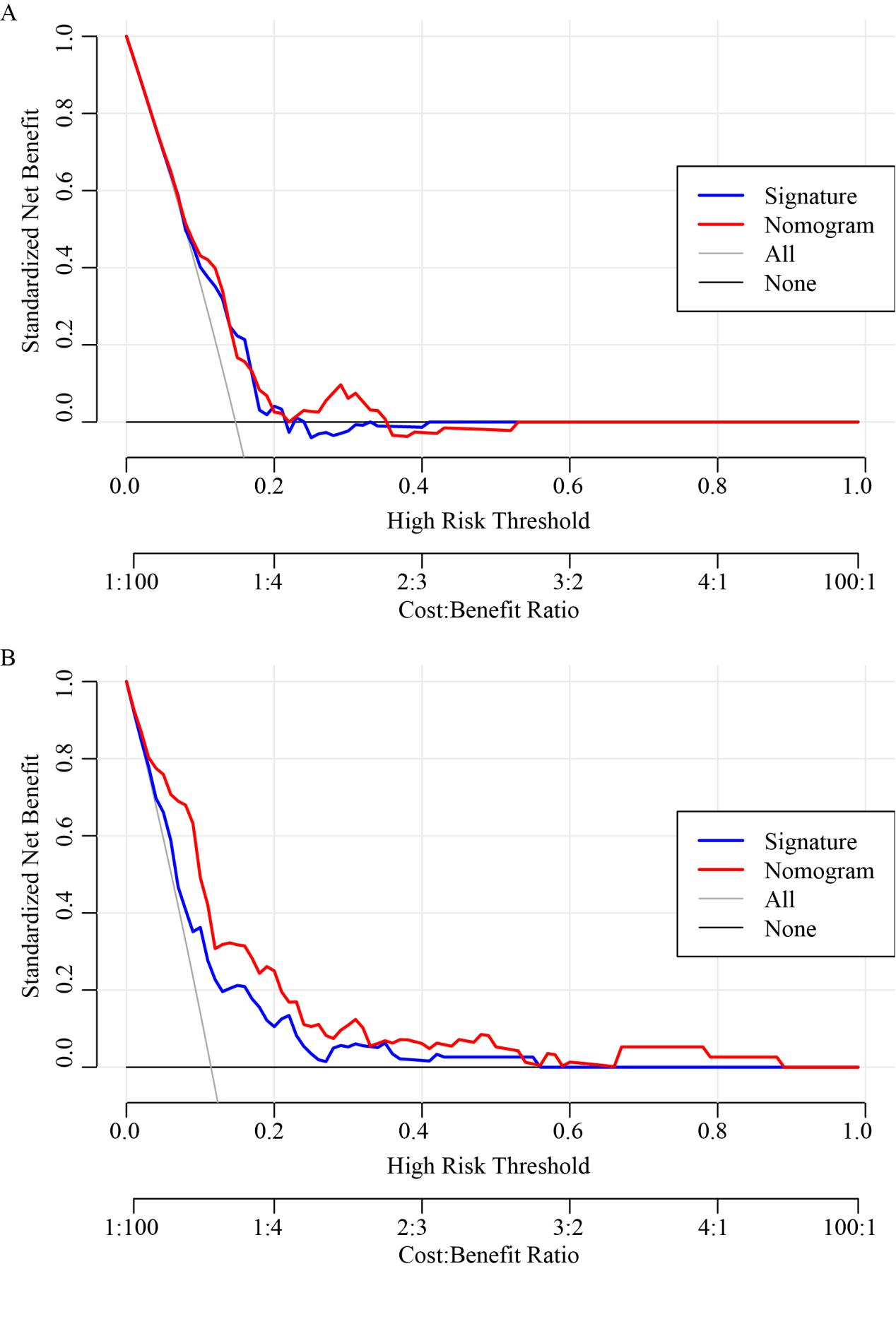
**

**Figure S12:** (A) Decision curve analysis of the nomogram and signature for the relapse-free survival prediction of stage I-III CRC patients in TCGA cohort. (B) Decision curve analysis of the nomogram and signature for the overall survival prediction of stage I-III CRC patients in TCGA cohort.

| **Table S6.** Primers used for PCR validation | |
| --- | --- |
| Gene | Forward and Reverse primer |
| MEST | F: 5' ATCGGGTGATTGCCCTTGATT 3' |
|  | R: 5' GAAAGAAGGTTGATCCTGCGG 3' |
| PLOD3 | F: 5' GACCCGGTCAACCCAGAGA 3' |
|  | R: 5' CTCCACCAACTGTTCGAGCC 3' |
| CXCL1 | F: 5' TCCTGCATCCCCCATAGTTA 3' |
|  | R: 5' CTTCAGGAACAGCCACCAGT 3' |
| FUCA1 | F: 5' GAAGCCAAGTTCGGGGTGTT 3' |
|  | R: 5' GGGTAGTTGTCGCGCATGA 3' |
| IL15 | F: 5' TTGGGAACCATAGATTTGTGCAG 3' |
|  | R: 5' GGGTGAACATCACTTTCCGTAT 3' |
| DST | F: 5' CTACCAGCACTCGAACCAGTC 3' |
|  | R: 5' GCCGAAGCTAATGCAAGAGTTG 3' |
| NT5E | F: 5' GCCTGGGAGCTTACGATTTTG 3' |
|  | R: 5' TAGTGCCCTGGTACTGGTCG 3' |
| LAMC2 | F: 5' CAAAGGTTCTCTTAGTGCTCGAT 3' |
|  | R: 5' CACTTGGAGTCTAGCAGTCTCT 3' |
| DKK1 | F: 5' CCTTGAACTCGGTTCTCAATTCC 3' |
|  | R: 5' CAATGGTCTGGTACTTATTCCCG 3' |
| PLOD2 | F: 5' CATGGACACAGGATAATGGCTG 3' |
|  | R: 5' AGGGGTTGGTTGCTCAATAAAAA 3' |
| ITGB1 | F: 5' GTAACCAACCGTAGCAAAGGA 3' |
|  | R: 5' TCCCCTGATCTTAATCGCAAAAC 3' |
| GJA1 | F: 5' GGTGACTGGAGCGCCTTAG 3' |
|  | R: 5' GCGCACATGAGAGATTGGGA 3' |
| ACTA2 | F: 5' AAAAGACAGCTACGTGGGTGA 3' |
|  | R: 5' GCCATGTTCTATCGGGTACTTC 3' |
| DPYSL3 | F: 5' GACCGTCTCCTTATCAAGGGA 3' |
|  | R: 5' GCATCTGGAAGTGAGTATGGAC 3' |
| CXCL12 | F: 5' ATTCTCAACACTCCAAACTGTGC 3' |
|  | R: 5' ACTTTAGCTTCGGGTCAATGC 3' |
| LAMA2 | F: 5' TGCTGTCCTGAATCTTGCTTC 3' |
|  | R: 5' AGCATTTGTAATCGGGTGTCTC 3' |
| MAGEE1 | F: 5' ACCAAGCACTTCCGTACTGC 3' |
|  | R: 5' GAGGTGTCCGATCCATCAGAG 3' |
| CRLF1 | F: 5' CCCAGAGAAACCCGTCAACAT 3' |
|  | R: 5' ACTGTGTGGTACTCCTCACAT 3' |
| CDH6 | F: 5' AGAACTTACCGCTACTTCTTGC 3' |
|  | R: 5' TGCCCACATACTGATAATCGGA 3' |

| **Table S7.** Baseline characteristics of CRC patients in FUSCC validation cohort. | |
| --- | --- |
| Variables | FUSCC cohort |
| Age (mean, range) | 61.1 (47-79) |
| Gender |  |
| Male | 58 |
| Female | 46 |
| T stage |  |
| T1 | 3 |
| T2 | 8 |
| T3 | 78 |
| T4 | 15 |
| N stage |  |
| N0 | 55 |
| N1 | 27 |
| N2 | 22 |
| Adjuvant Therapy |  |
| No | 9 |
| Yes | 87 |
| NA | 8 |
| KRAS status |  |
| Wild Type | 43 |
| Mutation | 30 |
| NA | 31 |
| Tumor location |  |
| Colon | 42 |
| Rectum | 62 |
| Total | 104 |
| CRC, colorectal cancer; FUSCC, Fudan University Shanghai Cancer Center | |

| **Table S8.** Univariable and multivariable Cox regression model analyses of relapse-free survival in GSE39582 cohort | | | | | | | |
| --- | --- | --- | --- | --- | --- | --- | --- |
| Variables | Univariable analysis | | |  | Multivariable analysis | | |
|  | HR | 95% CI | P-value |  | HR | 95% CI | P-value |
| Gender |  |  | 0.107 |  |  |  |  |
| Female | 1 |  |  |  |  |  |  |
| Male | 1.538 | 0.936-1.969 |  |  |  |  |  |
| Age |  |  | 0.247 |  |  |  |  |
| Mean±SD | 1.008 | 0.994-1.023 |  |  |  |  |  |
| Tumor site |  |  | 0.697 |  |  |  |  |
| Proximal | 1 |  |  |  |  |  |  |
| Distal | 1.038 | 0.862-1.250 |  |  |  |  |  |
| Chemotherapy |  |  | 0.002 |  |  |  | 0.711 |
| No | 1 |  |  |  | 1 |  |  |
| Yes | 1.799 | 1.251-2.585 |  |  | 0.960 | 0.772-1.193 |  |
| T stage |  |  | 0.018 |  |  |  | 0.048 |
| T1 | 1 |  |  |  | 1 |  |  |
| T2 | 3424 | 0-4.03E+50 | 0.883 |  | 3656 | 0-1.42E+51 | 0.883 |
| T3 | 8341 | 0-9.80E+50 | 0.870 |  | 7986 | 0-3.09E+51 | 0.872 |
| T4 | 13848 | 0-1.63E+51 | 0.863 |  | 12666 | 0-4.90E+51 | 0.866 |
| LNM |  |  | <0.001 |  |  |  | <0.001 |
| Negative | 1 |  |  |  | 1 |  |  |
| Positive | 2.489 | 1.721-3.600 |  |  | 2.206 | 1.425-3.416 |  |
| MMR status |  |  | 0.057 |  |  |  |  |
| pMMR | 1 |  |  |  |  |  |  |
| dMMR | 0.729 | 0.527-1.009 |  |  |  |  |  |
| TP53 status |  |  | 0.296 |  |  |  |  |
| Wild-type | 1 |  |  |  |  |  |  |
| Mutation | 1.124 | 0.903-1.401 |  |  |  |  |  |
| KRAS status |  |  | 0.024 |  |  |  | 0.081 |
| Wild-type | 1 |  |  |  | 1 |  |  |
| Mutation | 1.242 | 1.029-1.498 |  |  | 1.085 | 0.797-1.416 |  |
| HR, Hazard ratio; CI, Confidence interval; SD, Standard deviation; MMR, Mismatch repair | | | | | | | |

| **Table S9.** Univariable and multivariable Cox regression model analyses of overall survival in GSE39582 cohort | | | | | | | |
| --- | --- | --- | --- | --- | --- | --- | --- |
| Variables | Univariable analysis | | |  | Multivariable analysis | | |
|  | HR | 95% CI | P-value |  | HR | 95% CI | P-value |
| Gender |  |  | 0.065 |  |  |  | 0.007 |
| Female | 1 |  |  |  | 1 |  |  |
| Male | 1.399 | 0.979-1.998 |  |  | 1.627 | 1.133-2.338 |  |
| Age |  |  | <0.001 |  |  |  | <0.001 |
| Mean±SD | 1.042 | 1.026-1.059 |  |  | 1.048 | 1.031-1.066 |  |
| Tumor site |  |  | 0.310 |  |  |  |  |
| Proximal | 1 |  |  |  |  |  |  |
| Distal | 0.913 | 0.765-1.089 |  |  |  |  |  |
| Chemotherapy |  |  | 0.287 |  |  |  |  |
| No | 1 |  |  |  |  |  |  |
| Yes | 1.102 | 0.921-1.318 |  |  |  |  |  |
| T stage |  |  | 0.012 |  |  |  | 0.004 |
| T1 | 1 |  |  |  | 1 |  |  |
| T2 | 1.741 | 0.210-14.468 | 0.608 |  | 1.537 | 0.185-12.790 | 0.691 |
| T3 | 2.589 | 0.361-18.598 | 0.344 |  | 1.837 | 0.254-13.288 | 0.547 |
| T4 | 4.574 | 0.626-33.409 | 0.134 |  | 3.703 | 0.504-27.227 | 0.198 |
| LNM |  |  | 0.041 |  |  |  | 0.029 |
| Negative | 1 |  |  |  | 1 |  |  |
| Positive | 1.437 | 1.015-2.036 |  |  | 1.483 | 1.042-2.111 |  |
| MMR status |  |  | 0.644 |  |  |  |  |
| pMMR | 1 |  |  |  |  |  |  |
| dMMR | 1.063 | 0.820-1.377 |  |  |  |  |  |
| TP53 status |  |  | 0.211 |  |  |  |  |
| Wild-type | 1 |  |  |  |  |  |  |
| Mutation | 1.147 | 0.925-1.421 |  |  |  |  |  |
| KRAS status |  |  | 0.096 |  |  |  | 0.148 |
| Wild-type | 1 |  |  |  | 1 |  |  |
| Mutation | 1.164 | 0.973-1.393 |  |  | 1.378 | 0.933-2.037 |  |
| HR, Hazard ratio; CI, Confidence interval; SD, Standard deviation; MMR, Mismatch repair | | | | | | | |

| **Table S10.** Univariable and multivariable Cox regression model analyses of relapse-free survival in TCGA cohort | | | | | | | |
| --- | --- | --- | --- | --- | --- | --- | --- |
| Variables | Univariable analysis | | |  | Multivariable analysis | | |
|  | HR | 95% CI | P-value |  | HR | 95% CI | P-value |
| Gender |  |  | 0.336 |  |  |  |  |
| Female | 1 |  |  |  |  |  |  |
| Male | 1.324 | 0.747-2.348 |  |  |  |  |  |
| Age |  |  | 0.584 |  |  |  |  |
| Mean±SD | 1.007 | 0.983-1.031 |  |  |  |  |  |
| Tumor site |  |  | 0.114 |  |  |  |  |
| Colon | 1 |  |  |  |  |  |  |
| Rectum | 1.624 | 0.890-2.963 |  |  |  |  |  |
| T stage |  |  | 0.040 |  |  |  | 0.066 |
| T1 | 1 |  |  |  | 1 |  |  |
| T2 | 1.419 | 0.164-12.259 | 0.751 |  | 1.429 | 0.165-12.382 | 0.746 |
| T3 | 2.017 | 0.276-14.753 | 0.489 |  | 1.559 | 0.210-11.574 | 0.664 |
| T4 | 5.815 | 0.709-47.725 | 0.101 |  | 4.616 | 0.551-38.685 | 0.159 |
| N stage |  |  | 0.001 |  |  |  | 0.002 |
| N0 | 1 |  |  |  | 1 |  |  |
| N1 | 1.506 | 0.744-3.050 | 0.255 |  | 1.349 | 0.658-2.766 | 0.414 |
| N2 | 4.010 | 1.960-8.205 | <0.001 |  | 3.862 | 1.841-8.102 | <0.001 |
| Venous invasion |  |  | 0.262 |  |  |  |  |
| Negative | 1 |  |  |  |  |  |  |
| Positive | 1.473 | 0.748-2.898 |  |  |  |  |  |
| HR, Hazard ratio; CI, Confidence interval; SD, Standard deviation | | | | | | | |

| **Table S11.** Univariable and multivariable Cox regression model analyses of overall survival in TCGA cohort | | | | | | | |
| --- | --- | --- | --- | --- | --- | --- | --- |
| Variables | Univariable analysis | | |  | Multivariable analysis | | |
|  | HR | 95% CI | P-value |  | HR | 95% CI | P-value |
| Gender |  |  | 0.268 |  |  |  |  |
| Female | 1 |  |  |  |  |  |  |
| Male | 1.448 | 0.753-2.785 |  |  |  |  |  |
| Age |  |  | <0.001 |  |  |  | <0.001 |
| Mean±SD | 1.059 | 1.028-1.090 |  |  | 1.061 | 1.031-1.093 |  |
| Tumor site |  |  | 0.374 |  |  |  |  |
| Colon | 1 |  |  |  |  |  |  |
| Rectum | 0.671 | 0.278-1.616 |  |  |  |  |  |
| T stage |  |  | 0.004 |  |  |  | 0.024 |
| T1 | 1 |  |  |  | 1 |  |  |
| T2 | 8918 | 0-2.175E+88 | 0.927 |  | 3156 | 0-2.439E+67 | 0.915 |
| T3 | 14136 | 0-3.437E+88 | 0.923 |  | 3511 | 0-2.704E+67 | 0.913 |
| T4 | 62141 | 0-1.513E+89 | 0.911 |  | 13857 | 0-1.069E+68 | 0.899 |
| N stage |  |  | 0.006 |  |  |  | 0.006 |
| N0 | 1 |  |  |  | 1 |  |  |
| N1 | 1.538 | 0.699-3.388 | 0.285 |  | 1.883 | 0.817-4.339 | 0.137 |
| N2 | 3.613 | 1.635-7.986 | 0.001 |  | 4.047 | 1.719-9.531 | 0.001 |
| Venous invasion |  |  | 0.707 |  |  |  |  |
| Negative | 1 |  |  |  |  |  |  |
| Positive | 1.171 | 0.513-2.673 |  |  |  |  |  |
| HR, Hazard ratio; CI, Confidence interval; SD, Standard deviation | | | | | | | |

**Table S12**

Standardized net benefit using the overall survival nomogram based on GSE39582 for specific optimal thresholds.

| Threshold | Standardized net benefit all | Standardized net benefit signature | Standardized net benefit nomogram |
| --- | --- | --- | --- |
| 0.1 | 0.707 | 0.697 | 0.733 |
| 0.2 | 0.34 | 0.361 | 0.443 |
| 0.3 |  | 0.167 | 0.288 |
| 0.4 |  | 0.133 | 0.148 |
| 0.5 |  | 0.031 | 0.07 |
| 0.6 |  | 0.016 | 0.039 |
| 0.7 |  | 0 | 0.018 |
| 0.8 |  |  | -0.031 |
| 0.9 |  |  | 0 |

Standardized net benefit using the relapse-free survival nomogram based on GSE39582 for specific optimal thresholds.

| Threshold | Standardized net benefit all | Standardized net benefit signature | Standardized net benefit nomogram |
| --- | --- | --- | --- |
| 0.1 | 0.607 | 0.675 | 0.622 |
| 0.2 | 0.115 | 0.394 | 0.448 |
| 0.3 |  | 0.271 | 0.259 |
| 0.4 |  | 0.15 | 0.132 |
| 0.5 |  | 0.085 | 0.11 |
| 0.6 |  | 0.004 | 0.005 |
| 0.7 |  | 0.042 | -0.018 |
| 0.8 |  | 0.017 | 0.011 |
| 0.9 |  | 0 | 0 |

Standardized net benefit using the overall survival nomogram based on TCGA for specific optimal thresholds.

| Threshold | Standardized net benefit all | Standardized net benefit signature | Standardized net benefit nomogram |
| --- | --- | --- | --- |
| 0.1 | 0.14 | 0.363 | 0.491 |
| 0.2 |  | 0.105 | 0.25 |
| 0.3 |  | 0.053 | 0.109 |
| 0.4 |  | 0.018 | 0.061 |
| 0.5 |  | 0 | 0.053 |
| 0.6 |  |  | 0.013 |
| 0.7 |  |  | 0.053 |
| 0.8 |  |  | 0.026 |
| 0.9 |  |  | 0 |

Standardized net benefit using the relapse-free survival nomogram based on TCGA for specific optimal thresholds.

| Threshold | Standardized net benefit all | Standardized net benefit signature | Standardized net benefit nomogram |
| --- | --- | --- | --- |
| 0.1 | 0.358 | 0.401 | 0.431 |
| 0.2 |  | 0.041 | 0.026 |
| 0.3 |  | -0.023 | 0.061 |
| 0.4 |  | -0.014 | -0.027 |
| 0.5 |  | 0 | -0.02 |
| 0.6 |  |  | 0 |
